# Categorization of Bacteria That Leak from Activated Sludge to Secondary Treated Water: Year-round Observations

**DOI:** 10.1264/jsme2.ME24082

**Published:** 2025-03-15

**Authors:** Egodaha G. W. Gunawardana, Tiffany Joan Sotelo, Kenshiro Oshima, Masahira Hattori, Takashi Mino, Hiroyasu Satoh

**Affiliations:** 1 Department of Socio-Cultural Environmental Studies, Graduate School of Frontier Sciences, The University of Tokyo, 5–1–5 Kashiwanoha, Kashiwa, Chiba 277–8563, Japan; 2 Institute of Chemistry, College of Science, University of the Philippines Diliman, Diliman, Quezon City 1101, Philippines; 3 Center for Omics and Bioinformatics, Graduate School of Frontier Sciences, The University of Tokyo, 5–1–5 Kashiwanoha, Kashiwa, Chiba 277–8561, Japan

**Keywords:** activated sludge, secondary treated water, leak type, 16S rRNA, *Patescibacteria*

## Abstract

The present study proposes a categorization of bacteria that leak from activated sludge processes to secondary treated water (STW). Bacterial populations in primary treated water (PTW), activated sludge (AS), STW, and the 0.2‍ ‍μm-filtrate of STW (FSTW) in a full-scale wastewater treatment plant with two treatment trains were observed for a period of one year using a 16S rRNA ana­lysis approach. The taxonomic groups detected were categorized as different “leak types” based on the read occupancies in PTW, AS, STW, and FSTW, where a leak type indicates the likelihood of a taxonomic group to leak to STW. Five leak types were introduced: “LTE”, “LTE-I”, “LTEF”, “LTF”, and “NLT”, with “LT” for leak type, “E” for high read occupancy in STW or the effluent of secondary settling tanks, “I” for high read occupancy in PTW or influent to the AS process, “F” for high read occupancy in FSTW, and “NLT” for a smaller likelihood to leak. Representative taxonomic groups for each leak type were *Neisseria* and ABY1 for “LTE” *Parcubacteria* for “LTEF”, *Campylobacterota* for “LTE-I”, and *Saccharimonadia*, *Bdellovibrionota*, and some lineages in *Comamonadaceae* for “LTF”. Although some taxonomic groups, such as *Comamonadaceae*, included different leak types, the categorization assigned to each taxonomic group was mostly consistent between the two treatment trains. The categorization scheme proposed herein may become a useful key for understanding the characteristics of bacteria that appear in AS and STW.

Knowledge of the microbial populations in biological wastewater treatment processes is rapidly increasing due to the development of sequencing and bioinformatic technologies. For example, MiDAS (Dueholm *et al.*, 2022) organized by the Center for Microbial Community, Aalborg University, is a project that catalogues microorganisms in activated sludge (AS) and anaerobic digestion processes and connects physiological characteristics and phylogenetic identities with the operational conditions of the processes. Categorization keys are used in the collection of information on microbial populations. In MiDAS, microorganisms in AS are classified and categorized not only from a phylogenetic point of view, but also their cell properties and metabolism. The cell property categorization key includes whether the organism is filamentous and the cell surface is hydrophobic. Metabolic categorization keys include those related to nitrification, denitrification, and polyphosphate accumulation. The microorganisms in AS are responsible for wastewater treatment, and although some have useful functions for treatment, others cause problems, such as bulking. Therefore, the categorization of microorganisms in AS and a more detailed understanding of the AS ecosystem are useful for wastewater treatment.

Microbial populations in secondary treated water (STW) or wastewater treated by AS processes are also of interest because STW is the product of wastewater treatment. Gunawardana *et al.* (2014) demonstrated that the microbial population in STW differed from that in AS. Previous studies exami­ned microbial populations in STW (Tang *et al.*, 2016; Ahmed *et al.*, 2017; Do *et al.*, 2019; Numberger *et al.*, 2019; Xue *et al.*, 2019; Kristensen *et al.*, 2020; Pascual-Benito *et al.*, 2020; Newton *et al.*, 2022). These studies mainly focused on investigating health-related or potentially pathogenic microorganisms present in both primary treated water (PTW) and STW. However, microorganisms in STW are of interest because they may have an impact on the water environment in receiving water bodies or affect the performance of advanced treatment for reclaimed water use. Furthermore, microbial populations in STW may reflect the stability of microbial population structures in AS. Therefore, the further accumulation of knowledge of microbial populations in STW is of importance and the categorization of microorganisms in STW is necessary for this purpose.

A number of keys are used to categorize microorganisms in STW. The first key is categorization by origin: PTW or AS. Some microorganisms from PTW may survive the treatment process in AS and subsequently enter STW or otherwise may originate in AS. The second key is the likelihood of microorganisms originating from AS to leak to STW: some may be strongly associated with AS flocs and seldom leak to STW, while others may have be more likely to leave AS to STW. The third key is size. A group of extremely small, potentially parasitic bacteria was recently discovered. These bacteria, classified as the phylum *Patescibacteria* (Brown *et al.*, 2015; Parks *et al.*, 2018; Wiegand *et al.*, 2021), pass through 0.2-μm membrane filters and have been detected in various environments, including sewage treatment plants. They may be present in STW and are reportedly parasitic (Brown *et al.*, 2015; Parks *et al.*, 2018; Wiegand *et al.*, 2021), and may affect bacterial populations in AS. Therefore, their distribution in STW is of interest. Consequently, detecting these bacteria in the 0.2-μm filtrate of STW (FSTW) may be an additional categorization criterion.

By using these keys, namely, the source, likelihood to leave AS, and cell size, the present study aims to propose a method that categorizes bacteria in STW. To the best of our knowledge, this is the first study to propose the categorization of bacteria in STW using these keys. To implement the categorization, PTW, AS, STW, and FSTW samples were collected monthly for one year from two treatment trains at an urban wastewater treatment plant. Bacterial community compositions were analyzed using 16S rRNA amplicon sequencing. STW bacteria were categorized based on their relative abundance (read occupancy) in PTW, AS, STW, and FSTW.

## Materials and Methods

### AS, STW, and FSTW samples

All samples were collected from Mikawashima Water Reclamation Center, a full-scale urban sewage treatment plant in Tokyo, Japan. This sewage treatment plant receives sewage from combined sewer systems and has five treatment trains. Samples were obtained from Train A with the conventional AS process with reduced aeration in the upper stream aeration tanks and from Train B with the enhanced biological phosphorus removal AS process. Both trains use gravity settling to separate AS from water after treatment. The collected samples are tabulated in Table S1. Samples were obtained once a month for a period of one year between February 2010 and January 2011. Samples were also collected from Monday to Friday in one week in February, May, August, and November 2010. The operational and water quality data provided by the treatment plant are tabulated in Table S2.

Approximately 1.0 L of the AS mixture was taken at the end of the aeration tank and settled on-site for more than 30‍ ‍min but less than 2 h. Approximately 500‍ ‍mL of the supernatant was carefully collected and used as the STW sample in ana­lyses. STW samples were not taken from secondary settling tanks to avoid the effects of hydraulic loads caused by stormwater inflow. STW samples were carefully collected while avoiding disturbances in the sludge bed to prevent the collection of bulking-related flocs in STW samples.

Approximately 25‍ ‍mL of the remaining AS mixture was centrifuged at 3,500‍ ‍rpm for 5‍ ‍min. The supernatant was discarded and the pellet was re-suspended in 25‍ ‍mL Milli-Q water and used as the AS sample. Some of the STW sample was filtered through a 0.2-μm membrane filter (DISMIC 25AS020AN; Toyo Roshi Kaisha) to obtain the FSTW sample. Each of these samples was stored at –80°C until used.

A template DNA solution for a PCR ana­lysis of AS and STW samples was prepared by the sonication-dilution method (Satoh *et al.*, 2013; Gunawardana *et al.*, 2014) with minor modifications. Sonication was applied to 1.0‍ ‍mL of the AS sample, or the original STW sample was placed in a 2.0-mL plastic tube and sonicated with an Advanced-Digital Sonifier 250 (Branson) equipped with a microtip horn at an amplitude value of 30% for 20 s. Sonicated samples were used as the template for PCR without purification and were further diluted with autoclaved ultrapure water to make DNA concentrations in the PCR mixture in a range of 1.0 to 10.0‍ ‍pg‍ ‍μL^–1^. FSTW samples were used as the template for PCR without any purification or dilution. The template DNA concentrations of the extracted samples and FSTW samples were quantified using the Pico-Green dsDNA quantification kit (Invitrogen) according to the manufacturer’s instructions.

The partial 16S rRNA gene in prepared templates was amplified using the universal primer set of the 27f (5′-AGAGTTTGATCMTGGCTCAG-3′) and 519r primers (5′-GWATTACCGCGGCKGCTG-3′) (Lane, 1991), each with an 8-base barcode sequence on the 5′ end as shown in Table S1, with ExTaq Hot Start Version (Takara) as the DNA polymerase kit. The thermal program was as follows: at 95°C for 10‍ ‍min followed by 30 cycles at 94°C for 30‍ ‍s, 55.3°C for 30‍ ‍s, and 72°C for 30‍ ‍s, and a final extension step at 72°C for 10‍ ‍min.

### PTW samples

PTW samples were collected in the sampling campaigns in October and December 2010. Collected samples were ice-cooled during transport and then stored at –80°C in the laboratory until used.

PTW samples were sonicated and diluted if necessary with autoclaved ultrapure water in the same manner as AS and effluent samples. Amplification was performed using PrimeScript One Step PCR Kit Version 2 (Takara), which is a kit for reverse transcription PCR (RT-PCR) following the protocol reported by Satoh *et al.*, 2013. The thermal program was as follows: at 50°C for 30‍ ‍min followed by 94°C for 2‍ ‍min and 20 thermal cycles at 94°C for 30‍ ‍s, 55.3°C for 30‍ ‍s, and 72°C for 30‍ ‍s, and a final extension step at 72°C for 10‍ ‍min.

### Sequencing

PCR and RT-PCR products were purified with a High Pure PCR Clean Up Micro Kit (Roche). The quality of purified PCR products was exami­ned with a 2100 Bioanalyzer (Agilent) according to the manufacturer’s instructions. Purified PCR products from samples were mixed and sequenced using a Roche 454 FLX pyrosequencer. Raw sequences were deposited to the DDBJ Sequence Read Archive (DRA) under the project number PRJDB15930. Accession numbers are specified in Supplementary Table S1.

### Data ana­lysis

The sequences obtained were quality-checked and filtered by QIIME 1.9.1 (Caporaso *et al.*, 2010), further analyzed by QIIME2 (Bolyen *et al.*, 2019) with vsearch *de novo* clustering at 0.97 similarity, and taxonomy was assigned by uclust using the MiDAS 4.8.1 dataset (Dueholm *et al.*, 2022).

### Visualization of bacterial population structures

Product files from QIIME2, feature.qza (feature table, the matrix of read counts for each operational taxonomic unit (OTU) for each‍ ‍sample), and taxonomy.qza (taxonomy assignment for each OTU) were imported to OTUMAMiII (https://en.wwmlab.info/otumamiii-microbialpopulationvisualization), a database application developed on FileMaker Pro (Claris International) by the authors. For each sample for sample type *t* (PTW, AS, STW, or FSTW) for train *x* (A or B) on day *d*, the following were calculated by OTUMAMiII.

$$p_{OTU\_i,\;t,x,\;d}=\frac{n_{OTU\_i,\;t,x,d}}{N_{t,x,\;d}}＝\frac{n_{OTU\_i,\;t,x,d\;}}{\sum_{OTU\_i}n_{OTU\_i,\;t,x,d}}$$

$$p_{TG\_j,\;t,x,\;d}=\frac{\sum_{\left(OTU\_i\;in\;TG\_j\right)}n_{OTU\_i,\;t,x,d}}{N_{t,x,\;d}}$$

*max*(*p_OTUi_*)=maximum of *p_OTU_i,t,x,d_* throughout all samples

max(*p_TG_j_*)=maximum of *p_TG_j,t,x,d_* throughout all samples

*TG_j*: Taxonomic group *j* where a taxonomic group is at the phylum, class, order, family, or genus level.

*N_t,x,d_*: Number of reads for sample type *t* from train *x* on day *d*.

*n_OTU_i,t,x,d_*: Number of reads assigned to OTU *i* for sample type *t* from train *x* on day *d*.

*p_OTU_i,t,x,d_*: Read occupancy of OTU *i* for sample type *t* from train *x* on day *d*.

*p_TG_j,t,x,d_*: Read occupancy of taxonomic group *j* for sample type *t* from train *x* on day *d*.

The following aggregated proportions of OTUs and taxonomic groups were then calculated for train *x* (A or B) for sample type *t* (PTW, AS, STW, or FSTW).

$$P_{OTU\_i,\;t,x}=\frac{\sum_{d}n_{OTU\_i,\;t,x,d}}{\sum_{d}N_{t,x,\;d}}$$

$$P_{TG\_j,\;t,x}=\frac{\sum_{d}\left(\sum_{\left(OTUi\;in\;TG\_j\right)}n_{OTUi,\;t,x,d}\right)}{\sum_{d}N_{t,x,\;d}}$$

*P_OTUi,t,x_*: Read occupancy of OTU *i* for sample type *t* from train *x* aggregated by day.

*P_TG_j,t,x_*: Read occupancy of taxonomic group *j* for sample type *t* from train *x* aggregated by day.

**Fig. 1.**
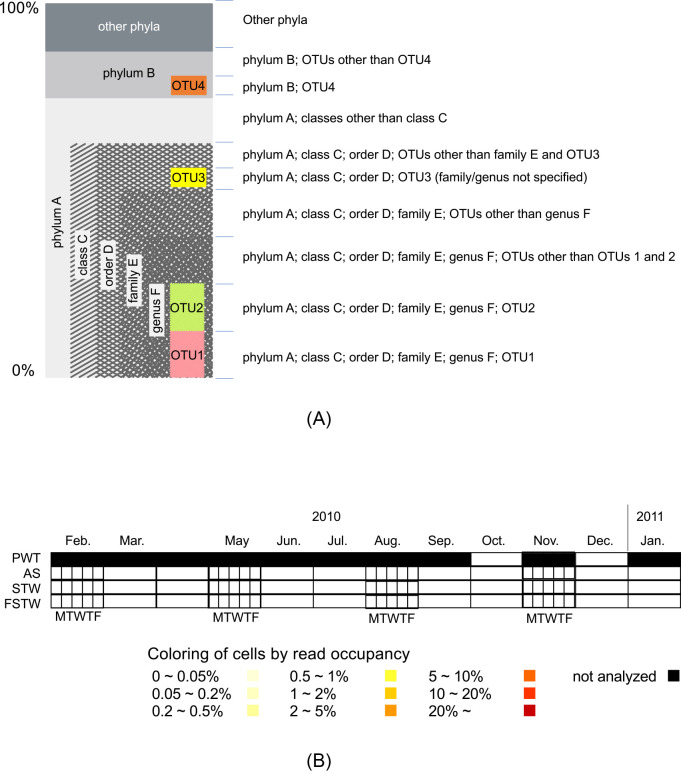
Structures of graphical representations of data: (A) hierarchically nested bar chart, and (B) two dimensional heatmap.

The bacterial population structures in each sample type of PTW, AS, STW, and FSTW in Trains A and B were visualized as follows using *max*(*p_TG_j_*) and max(*p_OTU_i_*). At each level of phylum, class, order, family, genus, and OTU, *max*(*p_TG_j_*)≥0.05 (for levels other than OTU) or *max*(*p_OTU_i_*)≥0.05 (for the OTU level) were extracted. The bacterial population in each sample type in each train was represented as a hierarchically nested bar chart, as shown in Fig. 1A. Data and drawing functions for visualizations were prepared by OTUMAMiII, and visualizations were performed using R (R Core Team, 2021). The source of reads detected in FSTW may originate not only from the living cells of extremely small bacteria, but also from DNA molecules released from damaged cells. Therefore, the composition of reads in FSTW samples should be called “composition of the 16S rRNA gene”. However, for simplicity, the read composition in FSTW was referred to as “bacterial population” in the present study.

The distributions of the selected OTUs and taxonomic groups in different samples were visualized using the “superheatmap” page of OTUMAMiII. The superheatmap page enables the creation of two-dimensional heatmaps using the repeating field objects of FileMaker Pro. The temporal distributions of the selected OTUs across different sample types were visualized as two-dimensional heat maps with an arrangement shown in Fig. 1B.

### Categorization of taxonomic groups based on their likelihood to leak to STW

To evaluate the likelihood of a taxonomic group to leak to STW, two types of leak type indexes defined below were calculated for taxonomic groups of interest.

LI1=1 (PTG, AS,x=0, PTG, STW,x=0)10 (PTG, AS,x=0, PTG, STW,x≠0)PTG, STW,xPTG, AS,x (PTG, AS,x≠0)

LI2=1 (PTG, STW,x=0, PTG, FSTW,x=0)10 (PTG, STW,x=0, PTG, FSTW,x≠0)PTG, FSTW,xPTG, STW,x (PTG, STW,x≠0)

*P_TG,AS,x_*: Read proportion of the taxonomic group of interest for AS in train *x*.

*P_TG,STW,x_*: Read proportion of the taxonomic group of interest for STW in train *x*.

*P_TG,FSTW,x_*: Read proportion of the taxonomic group of interest for FSTW in train *x*.

*LI*_1_ and *LI*_2_ are the ratios of the read proportions of a taxonomic group in STW to AS and FSTW to STW, respectively.

As an indicator of the source of the taxonomic group, the influent index below was defined and calculated.

II=1 (PTG, AS,x=0, PTG, PTW,x=0)10 (PTG, AS,x=0, PTG, PTW,x≠0)PTG, PTW,xPTG, AS,x (PTG, AS,x≠0)

*P_TG,PTW,x_*: Read proportion of the taxonomic group of interest for PTW in train *x*.

Taxonomic groups were then categorized as follows. While taking *C_a_* and *C_b_* as the threshold values, each taxonomic group was marked “LTE” (leak type with a likelihood to leak to effluent) when *LI*_1_>*C_a_* and *LI*_2_≤*C_b_*, “LTEF” (leak type with a likelihood to leak to effluent and filtered effluent) when *LI*_1_>*C_a_* and *LI*_2_>*C_b_*, “LTF” (leak type with a likelihood to leak to filtered effluent) when *LI*_1_≤*C_a_* and *LI*_2_>*C_b_*, or “NLT” (leak type with no likelihood to leak) when *P_TG,AS,x_*>*P_TG,STW,x_*>*P_TG,FSTW,x_*. When a taxonomic group did not fall into “LTE”, “LTEF”, “LTF”, or “NLT”, it was marked as “none”. When *II*≥*C_c_*, these marks were appended with “-I”, indicating a high possibility that the source is PTW rather than AS. In the present study, *C_a_*=2, *C_b_*=2, and *C_c_*=10 were used.

## Results

### General view

Template DNA concentrations in STW were in the range of 60 to 160‍ ‍μg L^–1^, while those in FSTW ranged between 25 to 35‍ ‍μg L^–1^. These STW and FSTW samples gave PCR products, except for two FSTW samples from Train A (Nov. 17 and Dec. 17, 2010). One of the STW samples from Train B was not analyzed for technical reasons. Therefore, reads were obtained from 169 samples. The number of effective reads obtained was 186,119, which consisted of 2,254 reads from PTW, 35,051 from AS, 89,244 from STW, and 59,569 from FSTW samples. Details on the number of reads from each sample are tabulated in Table S1.

**Fig. 2.**
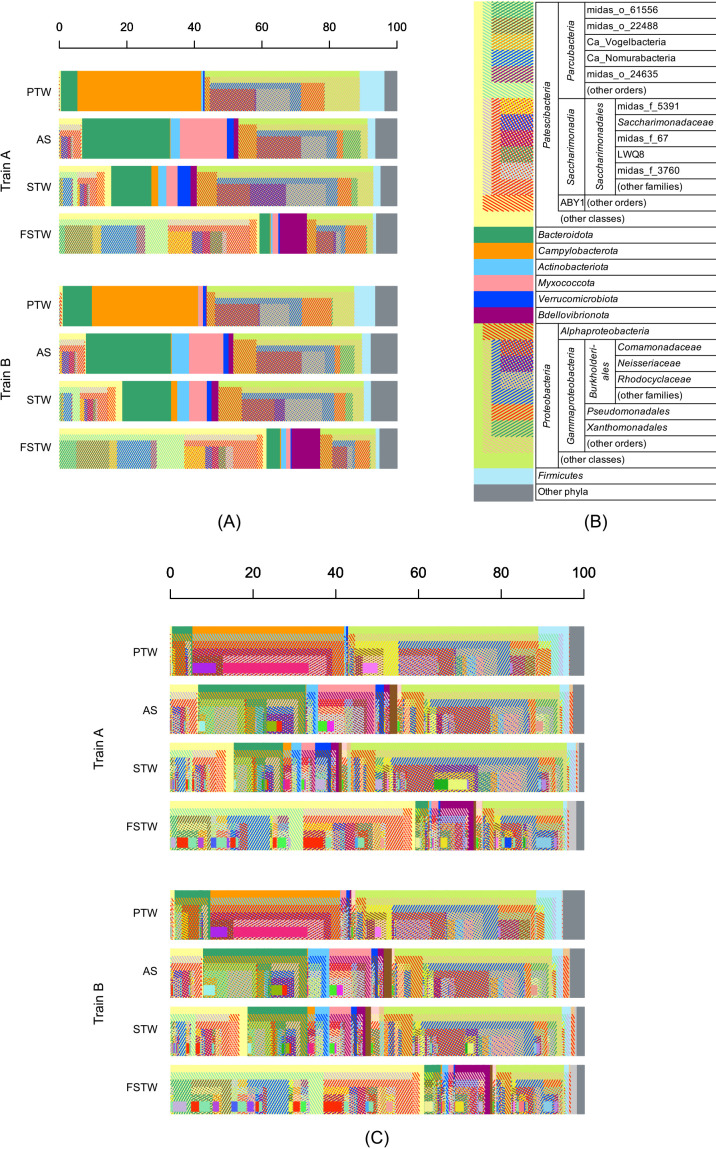
Hierarchically nested bar chart representations of read compositions for four types of samples from Trains A and B. (A) Simplified version, (B) the legend for (A), and (C) detailed version. The full legend for (C) is in Table S3.

The read occupancies of the respective taxonomic groups at different hierarchical levels in PTW, AS, STW, and FSTW from Trains A and B are presented as nested bar charts in Fig. 2A and C, where Fig. 2A is a simplified representation of Fig. 2C. Fig. 2B is the legend for Fig. 2A. The complete legend for Fig. 2C is shown in Table S3. Reads for PTW samples were obtained by RT-PCR, while reads for other samples were obtained by PCR. Although these two methods theoretically provide similar results, RT-PCR is considered to give more reads to active microorganisms with higher sensitivity (Heijnen *et al.*, 2024). In the present study, we disregard the possible differences caused by the differences between PCR or RT-PCR.

Similar bacterial population structures were observed between Trains A and B, as evidenced by similarities in the bar chart patterns of PTW, AS, STW, and FSTW for Trains A and B (Fig. 2). However, PTW, AS, STW, and FSTW significantly differed within each train. The solid thick lines in the bar charts in Fig. 2 represent OTUs, and OTU bars in the same color under each phylogenetic lineage represent an identical OTU. Many OTUs were commonly observed in these two trains, as shown in Fig. 2.

### Temporal dynamics

Samples were collected every month, making temporal dynamics a point of interest. However, the focus of the present study was categorization. The temporal dynamics observed in the two treatment trains with the four sample types, PTW, AS, STW, and FSTW were mainly used to show the consistency of observations.

**Fig. 3.**
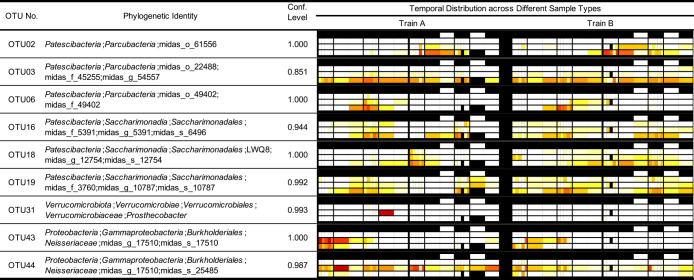
Phylogenetic identities and temporal distributions of OTUs collected across different sample types. The legend for the heatmaps is shown in Fig. 1B.

The temporal dynamics of OTUs, which occupied more than 5% of reads, at least in one of the 169 samples across different sample types, are shown in Table S4 together with their phylogenetic identities. A selection of 9 out of the 50 OTUs is presented in Fig. 3. The organization of heatmaps in Table S4 and Fig. 3 is illustrated in Fig. 1B.

Fig. 3 and Table S4 show that the temporal and sample type distributions of OTUs were similar in Trains A and B. While some OTUs were observed almost throughout the year (OTUs 03 and 44) in Trains A and B, other OTUs were only detected during a specific period (OTUs 02, 06, 16, 18, 19, and 43), with OTUs 16 and 18 being identified in two seasons. Some OTUs were observed sporadically in only one of the trains (OTU31).

### Leak type categorization

Table S5 shows the leak type categorization of all taxonomic groups with max(*p_TG_*)≥0.05 and OTUs with max(*p_OTU_*)≥0.05. Leak type categorizations for Trains A and B were identical in 183 of the 226 cases (81%) listed in Table S5. The frequencies of each leak type are listed in Table S5 and the taxonomic groups assigned to them are summarized in Table 1.

Table 1 shows that the dominant taxonomic groups in each leak type were the family *Neisseriaceae* in *Gammaproteobacteria*, followed by class ABY1 in *Patescibacteria* for LTE, *Parcubacteria* for LTEF, *Saccharimonadia* followed by *Bdellovibrionota* for LTF, and *Bacteroidota* and the family *Comamonadaceae* in *Gammaproteobacteria* for NLT. Some phyla included different leak types, with the phylum *Proteobacteria* showing a high diversity even at the genus level, as shown in Table S5. For example, *Comamonadaceae* included genera that were‍ ‍categorized as NLT (midas_g_33, midas_g_887, *Acidovorax*), LTE (midas_g_191), LTF or LTEF (*Aquabacterium*), or LTE-I/LTEF-I (*Malikia*).

## Discussion

The characteristics of each leak type are interpreted here. Bacteria in Group NLT were characterized by their preference to stay with other bacteria in AS, while those in Groups LTE and LTEF were mainly present in AS, but may‍ ‍have leaked to treated water. As its definition, bacteria in Group LTEF were more frequently detected in FSTW. Bacteria in Group LTE-I originate from PTW and survive through the treatment by AS. Although the DNA of bacteria in Group LTF were detected in FSTW, they were unlikely to leak to STW.

### LTE-I

As shown in Table 1 and Table S5, leak type LTE-I included *Campylobacterota* and three lineages in *Proteobacteria* in *Gammaproteobacteria*, namely, *Enterobacterales*, C39 in *Rhodocyclales*, and *Moraxellaceae* in* Pseudomonadales*, particularly *Acinetobacter*. Table 1 and Table S5 show that *Bacteroidales* in *Bacteroidota* was also categorized as LTE-I in Train B. Ahmed *et al.* (2017) detected genera in these groups in both raw sewage and STW: *Bacteroides* in *Bacteroidales*, *Arcobacter* in *Campylobacterota*, *Acinetobacter* in *Pseudomonadales*, and *Aeromonas* in *Enterobacterales*. The high abundance of *Arcobacter* in sewage and its leak to STW were previously reported (Xue *et al.*, 2019; Kristensen *et al.*, 2020; Newton *et al.*, 2022).

### LTEF and LTF

The taxonomic groups categorized as LTEF and LTF, for which DNA was detected at high frequencies in FSTW, are discussed here. *Patescibacteria* was the dominant group in these two leak types. The phylum *Patescibacteria* has very small genome and cell sizes and often lacks genes for enzymes that synthesize lipids, amino acids, and nucleic acids (Jaffe *et al.*, 2020). Due to these characteristics, they are generally considered to have a parasitic or symbiotic lifestyle (Wiegand *et al.*, 2021).

*Saccharimonadia*, also known as TM7, was mostly categorized under LTF. Bacteria in this group have been detected in filtrates from activated sludge mixed liquor (Batinovic *et al.*, 2021; Kagemasa *et al.*, 2022). However, previous studies on TM7 cells detected in AS by FISH (Hugenholtz *et al.*, 2001; Kindaichi *et al.*, 2016) detected a filamentous shape and similar sizes to usual bacteria. Cross *et al.* (2019) and Murugkar *et al.* (2020) reported the establishment of a co-culture of TM7 with actinobacterial host cells. Murugkar *et al.* (2020) discussed the discrepancy between the reported large cell sizes based on FISH images and the small genome sizes reported for TM7, which suggested the lack of many essential functional genes and indicated that the morphology reported by FISH was an error. In the present study, *Saccharimonadia* was detected with high read occupancies in AS, STW, and FSTW (Fig. 2). Collectively, these findings are consistent with *Saccharimonadia* having a very small cell size and also being detected by FISH in AS flocs.

*Parcubacteria*, also known as OD1, was categorized to LTEF. Bacteria in this group have been reported in groundwater filtrates (Nelson and Stegen, 2015; Brown *et al.*, 2015). Many *Parcubacteria* reads were found in FSTW in the present study, which is consistent with these findings. However, *Parcubacteria* reads occupied only approximately 0.15% of reads from AS, but accounted for around 35% in FSTW. Since the DNA concentration in AS is approximately 30–50‍ ‍mg L^–1^, while that in FSTW is around 20–50‍ ‍μg L^–1^, the weak detection of *Parcubacteria* reads in AS may be explained by this approximately 1,000× difference. However, this is contradictory to the observations on Saccharibacteria, which occupied approximately 7% of reads in AS and 23% in FSTW. While their reads were not detected in PTW in the present study, *Parcubacteria* may be coming from the influent. Further studies are needed to identify the source of *Parcubacteria* in FSTW.

The leak type LTEF also included the phylum *Bdellovibrionota*. This phylum was rarely detected in the influent, and occupied approximately 1.5% of reads in AS. These results suggest that this group is from AS. The high frequency of their detection in FSTW is unclear. The cell sizes of *Bdellovibrionota* are similar or smaller than those of conventionally known bacteria and may pass through a 0.45-μm filter, but may be removed with a 0.22-μm membrane filter (Sun *et al.*, 2017). Their DNA may have leaked outside of their cells or bacteria attacked by *Bdellovibrionota* may have an immune mechanism that resulted in the destruction of *Bdellovibrionota* cells.

In addition to *Parcubacteria* and *Bdellovibrionota*, the following were categorized to LTF or LTEF, as shown in Table S4: *Bacteroidota*; OC31 (particularly OTU20), *Actinobacteria*; *Thermoleophilia* (particularly OTU28), *Proteobacteria*; *Alphaproteobacteria*; *Rickettsiales*; AB1;‍ ‍midas_g_87794 (OTU36), *Proteobacteria*; *Gammaproteobacteria*; *Burkholderiales*; *Comamonadaceae*; *Aquabacterium* (OTU40,OTU41), and *Proteobacteria*; *Gammaproteobacteria*; *Pseudomonadales*; *Moraxellaceae*; *Agitococcus*_*lubricus*_group (OTU46). Bacteria in these groups are considered to have cell sizes larger than 0.45‍ ‍μm; therefore, it currently remains unclear why the DNA of these bacterial groups was detected in FSTW. OTU20 was also detected at significant levels in AS. On the other hand, OTU28, OTU36, OTU40, OTU41, and OTU46 were not or were rarely detected in AS and behaved more like *Parcubacteria*. The possible origin of the DNA for these reads is of interest. Regarding OTU20, DNA molecules may have been released in lysis by phages or ingestion by higher organisms. Including the case of OTU20, these may be related with parasitic bacteria or phages.

### LTE

Taxonomic groups categorized as LTE include *Neisseriaceae* in *Gammaproteobacteria*, ABY1 in *Patescibacteria*, some *Bacteroidota*, some *Verrucomicrobia*, Dependiae, and several lineages of *Proteobacteria*. Reads classified as *Neisseriaceae* were classified as *Procabacteria* using GreenGenes13.8. Of the six studies that investigated bacterial populations in treated water (Tang *et al.*, 2016; Ahmed *et al.*, 2017; Do *et al.*, 2019; Xue *et al.*, 2019; Kristensen *et al.*, 2020; Pascual-Benito *et al.*, 2020; Newton *et al.*, 2022), only Xue *et al.* (2019) detected *Procabacteria* in STW. Therefore, the predominance of *Neisseriaceae* in STW may not be universal and unique to the treatment plant studied here.

## Conclusions

In the present study, a scheme was proposed to categorize bacteria detected in STW. Their origin, PTW or AS, their likelihood to leave AS flocs, and their detection in FSTW were used as keys. The taxonomic groups detected were categorized for different “leak types” based on their read occupancies in PTW, AS, STW, and FSTW, where a leak type indicates the likelihood of a taxonomic group to leak to STW. Representative taxonomic groups for each leak type were as follows:

1. *Parcubacteria*, which were significantly detected in STW and FSTW than in AS, were categorized as LTEF.

2. *Campylobacterota*, which were significantly detected in both STW and PTW, were categorized as LTE-I.

3. *Neisseria* and ABY1, which were significantly detected in STW, but not in FSTW or PTW, were categorized as LTE.

4. *Saccharimonadia* and *Bdellovibrionota* and some lineages in *Comamonadaceae*, which were significantly detected in FSTW, but not in STW or PTW, were categorized as LTF.

5. Bacteria that were less likely to leak to STW and FSTW were categorized as NLT.

Although some taxonomic groups, such as *Comamonadaceae*, included different leak types, the categorization assigned to each taxonomic group was mostly consistent between the two treatment trains. The general applicability of the above listed conclusions needs to be exami­ned further in different wastewater treatment plants.

The categorization scheme proposed herein may be useful for characterizing microorganisms in AS and STW. This categorization is related to ecological characteristics, such as their capability to escape predation by AS microorganisms, their likelihood to leave AS flocs, and their potentially parasitic characteristics. A more detailed understanding of micro­organisms in STW will lead to the better management of water environments in receiving water bodies, improvements in the performance of advanced water treatment for reclaimed water use, and a more detailed understanding of microbial ecosystems in AS, which play a key role in wastewater treat­ment.

## Citation

Gunawardana, E. G. W., Sotelo, T. J., Oshima, K., Hattori, M., Mino, T., and Satoh, H. (2025) Categorization of Bacteria That Leak from Activated Sludge to Secondary Treated Water: Year-round Observations. *Microbes Environ ***40**: ME24082.

https://doi.org/10.1264/jsme2.ME24082

## Supplementary Material

Supplementary Material

## Figures and Tables

**Table 1.** Summary of leak type categorization results.

	LTE	LTE-I	LTEF	LTF	NLT	none
*Patescibacteria*						
*Parcubacteria*			XXXXXX			
*Saccharimonadia*			X	XXXXXX		
ABY1	XX					
*Bacteroidota*	X	X			XXXXXXXXXXXXX	
*Campylobacterota*		XX				
*Actinobacteriota*			—	—	XXX	
*Myxococcota*					XXXX	
*Verrucomicrobia*	X				X	
*Bdellovibrionota*			—	XX		
Planctomycetota					X	
*Spirochaeota*	X					X
*Dependentiae*	X					
*Proteobacteria*						
*Alphaproteobacteria*						
*Paracaedibacterales*	X					
*Rickettsiales*	X		—			
*Sphingomonadales*					X	
*Rhodobacterales*						X
*Gammaproteobacteria*						
*Competibacterales*	X				—	
*Enterobacterales*	—	XX				
*Burkholderiales*						
*Sutterellaceae*					X	
*Comamonadaceae*	—	—	—		XXXXXXXXX	
*Neisseriaceae*	XXXXXXX	—				
*Rhodocyclaceae*						
C39		XX				
Ca_Accumulibacter					X	X
*Dechloromonas*					X	
*Zoogloea*	XX				XX	
*Moraxellaceae*						
*Agitococcus*			X			
*Acinetobacter*	X	X				
*Xanthomonadales*					XX	
*Firmicutes*					XX	—
*Chloroflexi*					—	—

The number of “X” marks represents the read occupancy percentage in STW, and “—” represents existence at less than 1%.
