# Supplementary material for: Categorization of Bacteria That Leak from Activated Sludge to Secondary Treated Water: Year-round Observations
Source: Microbes Environ. 2025 Mar 15;40(1):ME24082. doi: 10.1264/jsme2.ME24082 (PMC11946410; doi:10.1264/jsme2.ME24082)
Supplement: Supplementary file 1 — Supplementary Material [file 40_24082_s1.pdf]

Supplemental Material

Categorization of Bacteria That Leak from Activated Sludge to Secondary Treated Water: Year-round Observations

Egodaha G.W. Gunawardana[a1], Tiffany Joan Sotelo[b], Kenshiro Oshima[c2], Masahira Hattori[c3], Takashi Mino[a4], Hiroyasu Satoh[a5]

a Department of Socio-Cultural Environmental Studies, Graduate School of Frontier Sciences, The University of Tokyo, 5-1-5 Kashiwa, Chiba 277-8563, Japan.
b Institute of Chemistry, College of Science, University of the Philippines Diliman, Diliman, Quezon City 1101, Philippines
c Center for Omics and Bioinformatics, Graduate School of Frontier Sciences, The University of Tokyo, 5-1-5 Kashiwa, Chiba 277-8561, Japan.

E-mail addresses: hiroyasu@edu.k.u-tokyo.ac.jp

Running header: Categorization of bacteria in STW

Present addresses

[1] China-Sri Lanka Joint Research and Demonstration Centre (JRDC) for Water Technology, Ministry of Water Supply, Sri Lanka (wasanagun@gmail.com)

[2] Laboratory of genomics for health and longevity, School of Pharmacy, Kitasato University, 5-9-1 Shirokane, Minato-ku, Tokyo 108-8641, Japan (oshimak@pharm.kitasato-u.ac.jp)

[3] Faculty of Science and Engineering, Graduate School of Advanced Science and Engineering, Waseda University, 3-4-1 Okubo, Shinjuku-ku, Tokyo 169-8555 JAPAN (hattori@edu.k.u-tokyo.ac.jp)

[4] Professor Emeritus, The University of Tokyo, Japan (mino@edu.k.u-tokyo.ac.jp)

[5] Corresponding author. Department of Socio-Cultural Environmental Studies, Graduate School of Frontier Sciences, The University of Tokyo, 5-1-5 Kashiwa, Chiba 277-8563, Japan. hiroyasu@edu.k.u-tokyo.ac.jp, tel: +81-4-7136-4816, fax: +81-4-7136-4826

Table S1 Samples, accession numbers, and effective read numbers.

Train A

	Influent				Activated Sludge				Secondary Treated Water				Filtered Secondary Treated Water				
Date	Lane	Barcode	BioSample Accession Number	Number of effective reads	Lane	Barcode	BioSample Accession Number	Number of effective reads	Lane	Barcode	BioSample Accession Number	Number of effective reads	Lane	Barcode	BioSample Accession Number	Number of effective reads	
Feb. 15, 2010	(sample not taken)				1	AGAGAGAG	SAMD00728365	639	2	AGAGAGAG	SAMD00728393	1,797	8	AGAGAGAG	SAMD00728421	425	
Feb. 16, 2010	(sample not taken)				1	AGAGATGC	SAMD00728366	359	2	AGAGATGC	SAMD00728394	1,124	8	AGAGATGC	SAMD00728422	476	
Feb. 17, 2010	(sample not taken)				1	AGAGCAGC	SAMD00728367	748	2	AGAGCAGC	SAMD00728395	1,332	8	AGAGCAGC	SAMD00728423	57	
Feb. 18, 2010	(sample not taken)				1	AGAGCATG	SAMD00728368	691	2	AGAGCATG	SAMD00728396	1,633	8	AGAGCATG	SAMD00728424	402	
Feb. 19, 2010	(sample not taken)				1	AGATCATC	SAMD00728369	483	2	AGATCATC	SAMD00728397	1,785	8	AGATCATC	SAMD00728425	369	
Mar. 12, 2010	(sample not taken)				1	AGATCTGC	SAMD00728370	503	2	AGATCTGC	SAMD00728398	2,609	8	AGATCTGC	SAMD00728426	575	
Apr. 23, 2010	(sample not taken)				1	AGATGAGC	SAMD00728371	770	2	AGATGAGC	SAMD00728399	1,865	8	AGATGAGC	SAMD00728427	495	
May 10, 2010	(sample not taken)				1	AGATGATG	SAMD00728372	208	2	AGATGATG	SAMD00728400	495	8	AGATGATG	SAMD00728428	424	
May 11, 2010	(sample not taken)				1	AGATGCAG	SAMD00728373	850	2	AGATGCAG	SAMD00728401	2,087	8	AGATGCAG	SAMD00728429	448	
May 12, 2010	(sample not taken)				1	AGATGCTC	SAMD00728374	873	2	AGATGCTC	SAMD00728402	1,407	8	AGATGCTC	SAMD00728430	645	
May 13, 2010	(sample not taken)				1	AGCAGAGC	SAMD00728375	910	2	AGCAGAGC	SAMD00728403	2,382	8	AGCAGAGC	SAMD00728431	752	
May 14, 2010	(sample not taken)				1	AGCAGATG	SAMD00728376	876	2	AGCAGATG	SAMD00728404	1,423	8	AGCAGATG	SAMD00728432	373	
Jun. 18, 2010	(sample not taken)				1	AGCAGCAG	SAMD00728377	320	2	AGCAGCAG	SAMD00728405	1,749	8	AGCAGCAG	SAMD00728433	711	
Jul. 9, 2010	(sample not taken)				1	AGCAGCTC	SAMD00728378	580	2	AGCAGCTC	SAMD00728406	1,096	8	AGCAGCTC	SAMD00728434	544	
Aug. 2, 2010	(sample not taken)				1	AGCATCTG	SAMD00728379	508	2	AGCATCTG	SAMD00728407	1,646	8	AGCATCTG	SAMD00728435	720	
Aug. 3, 2010	(sample not taken)				1	AGCATGAG	SAMD00728380	360	2	AGCATGAG	SAMD00728408	3,039	8	AGCATGAG	SAMD00728436	848	
Aug. 4, 2010	(sample not taken)				1	AGCTCAGC	SAMD00728381	733	2	AGCTCAGC	SAMD00728409	2,002	8	AGCTCAGC	SAMD00728437	555	
Aug. 5, 2010	(sample not taken)				1	AGCTCATG	SAMD00728382	658	2	AGCTCATG	SAMD00728410	1,806	8	AGCTCATG	SAMD00728438	527	
Aug. 6, 2010	(sample not taken)				1	AGCTGATC	SAMD00728383	763	2	AGCTGATC	SAMD00728411	1,115	8	AGCTGATC	SAMD00728439	1,247	
Sep. 3, 2010	(sample not taken)				1	AGCTGCTG	SAMD00728384	549	2	AGCTGCTG	SAMD00728412	1,183	8	AGCTGCTG	SAMD00728440	681	
Oct. 15, 2010	5	CAGATCTG	SAMD00728363	582	1	ATCAGATC	SAMD00728385	354	2	ATCAGATC	SAMD00728413	2,822	8	ATCAGATC	SAMD00728441	507	
Nov. 15, 2010	(sample not taken)				1	ATCAGCTG	SAMD00728386	440	2	ATCAGCTG	SAMD00728414	2,252	8	ATCAGCTG	SAMD00728442	561	
Nov. 16, 2010	(sample not taken)				1	ATCATCAG	SAMD00728387	241	2	ATCATCAG	SAMD00728415	2,374	8	ATCATCAG	SAMD00728443	397	
Nov. 17, 2010	(sample not taken)				1	ATCATCTC	SAMD00728388	254	2	ATCATCTC	SAMD00728416	2,040	PCR product not obtained				
Nov. 18, 2010	(sample not taken)				1	ATCTCATC	SAMD00728389	122	2	ATCTCATC	SAMD00728417	399	8	ATCTCATC	SAMD00728445	340	
Nov. 19, 2010	(sample not taken)				1	ATCTCTGC	SAMD00728390	427	2	ATCTCTGC	SAMD00728418	1,995	8	ATCTCTGC	SAMD00728446	399	
Dec. 17, 2010	5	CAGATGAG	SAMD00728364	503	1	ATCTGAGC	SAMD00728391	177	2	ATCTGAGC	SAMD00728419	1,128	PCR product not obtained				
Jan. 21, 2011	(sample not taken)				1	ATCTGATG	SAMD00728392	104	2	ATCTGATG	SAMD00728420	1,079	8	ATCTGATG	SAMD00728448	349	Total
subtotal				1,085				14,500				47,664				13,827	77,076

Train B

	Influent				Activated Sludge				Secondary Treated Water				Filtered Secondary Treated Water				
Date	Lane	Barcode	BioSample Accession Number	Number of effective reads	Lane	Barcode	BioSample Accession Number	Number of effective reads	Lane	Barcode	BioSample Accession Number	Number of effective reads	Lane	Barcode	BioSample Accession Number	Number of effective reads	
Feb. 15, 2010	(sample not taken)				1	ATCTGCAG	SAMD00728451	1,147	2	ATCTGCAG	SAMD00728479	1,596	8	ATCTGCAG	SAMD00728507	460	
Feb. 16, 2010	(sample not taken)				1	ATCTGCTC	SAMD00728452	591	2	ATCTGCTC	SAMD00728480	662	8	ATCTGCTC	SAMD00728508	581	
Feb. 17, 2010	(sample not taken)				1	ATGAGAGC	SAMD00728453	192	2	ATGAGAGC	SAMD00728481	3,035	8	ATGAGAGC	SAMD00728509	523	
Feb. 18, 2010	(sample not taken)				1	ATGAGATG	SAMD00728454	235	2	ATGAGATG	SAMD00728482	724	8	ATGAGATG	SAMD00728510	171	
Feb. 19, 2010	(sample not taken)				4	AGAGAGAG	SAMD00728455	417	5	AGAGAGAG	SAMD00728483	1,557	6	AGAGAGAG	SAMD00728511	1,952	
Mar. 12, 2010	(sample not taken)				4	AGAGATGC	SAMD00728456	1,804	5	AGAGATGC	SAMD00728484	983	6	AGAGATGC	SAMD00728512	1,158	
Apr. 23, 2010	(sample not taken)				4	AGAGCAGC	SAMD00728457	752	5	AGAGCAGC	SAMD00728485	2,142	6	AGAGCAGC	SAMD00728513	1,833	
May 10, 2010	(sample not taken)				4	AGAGCATG	SAMD00728458	1,357	5	AGAGCATG	SAMD00728486	1,242	6	AGAGCATG	SAMD00728514	1,811	
May 11, 2010	(sample not taken)				4	AGATCATC	SAMD00728459	300	5	AGATCATC	SAMD00728487	1,094	6	AGATCATC	SAMD00728515	1,947	
May 12, 2010	(sample not taken)				4	AGATCTGC	SAMD00728460	863	5	AGATCTGC	SAMD00728488	975	6	AGATCTGC	SAMD00728516	1,379	
May 13, 2010	(sample not taken)				4	AGATGAGC	SAMD00728461	227	5	AGATGAGC	SAMD00728489	1,325	6	AGATGAGC	SAMD00728517	2,278	
May 14, 2010	(sample not taken)				4	AGATGATG	SAMD00728462	662	5	AGATGATG	SAMD00728490	1,792	6	AGATGATG	SAMD00728518	1,633	
Jun. 18, 2010	(sample not taken)				4	AGATGCAG	SAMD00728463	649	5	AGATGCAG	SAMD00728491	3,228	6	AGATGCAG	SAMD00728519	1,997	
Jul. 9, 2010	(sample not taken)				4	AGATGCTC	SAMD00728464	608	5	AGATGCTC	SAMD00728492	1,473	6	AGATGCTC	SAMD00728520	2,087	
Aug. 2, 2010	(sample not taken)				4	AGCAGAGC	SAMD00728465	1,619	5	AGCAGAGC	SAMD00728493	891	6	AGCAGAGC	SAMD00728521	3,003	
Aug. 3, 2010	(sample not taken)				4	AGCAGATG	SAMD00728466	1,004	5	AGCAGATG	SAMD00728494	484	6	AGCAGATG	SAMD00728522	1,836	
Aug. 4, 2010	(sample not taken)				4	AGCAGCAG	SAMD00728467	422	not analyzed				6	AGCAGCAG	SAMD00728523	3,104	
Aug. 5, 2010	(sample not taken)				4	AGCAGCTC	SAMD00728468	292	5	AGCAGCTC	SAMD00728496	1,915	6	AGCAGCTC	SAMD00728524	1,727	
Aug. 6, 2010	(sample not taken)				4	AGCATCTG	SAMD00728469	617	5	AGCATCTG	SAMD00728497	1,892	6	AGCATCTG	SAMD00728525	2,786	
Sep. 3, 2010	(sample not taken)				4	AGCATGAG	SAMD00728470	681	5	AGCATGAG	SAMD00728498	1,836	6	AGCATGAG	SAMD00728526	2,654	
Oct. 15, 2010	5	CAGCAGAG	SAMD00728449	575	4	AGCTCAGC	SAMD00728471	916	5	AGCTCAGC	SAMD00728499	2,019	6	AGCTCAGC	SAMD00728527	1,794	
Nov. 15, 2010	(sample not taken)				4	AGCTCATG	SAMD00728472	997	5	AGCTCATG	SAMD00728500	1,949	6	AGCTCATG	SAMD00728528	1,547	
Nov. 16, 2010	(sample not taken)				4	AGCTGATC	SAMD00728473	1,089	5	AGCTGATC	SAMD00728501	2,311	6	AGCTGATC	SAMD00728529	1,994	
Nov. 17, 2010	(sample not taken)				4	AGCTGCTG	SAMD00728474	1,506	5	AGCTGCTG	SAMD00728502	607	6	AGCTGCTG	SAMD00728530	855	
Nov. 18, 2010	(sample not taken)				4	ATCAGATC	SAMD00728475	225	5	ATCAGATC	SAMD00728503	1,354	6	ATCAGATC	SAMD00728531	1,040	
Nov. 19, 2010	(sample not taken)				4	ATCAGCTG	SAMD00728476	471	5	ATCAGCTG	SAMD00728504	2,291	6	ATCAGCTG	SAMD00728532	1,272	
Dec. 17, 2010	5	CAGCATGC	SAMD00728450	595	4	ATCATCAG	SAMD00728477	649	5	ATCATCAG	SAMD00728505	1,418	6	ATCATCAG	SAMD00728533	1,182	
Jan. 21, 2011	(sample not taken)				4	ATCATCTC	SAMD00728478	259	5	ATCATCTC	SAMD00728506	785	6	ATCATCTC	SAMD00728534	1,138	Total
subtotal				1,170				20,551				41,580				45,742	109,043

					Total
Total		2,255	35,051	89,244	59,569	186,119

Table S2 Data provided by the WWTP. The figures are the monthly average values.

			2010											2011
			Feb.	Mar.	Apr.	May	Jun.	Jul.	Aug.	Sep.	Oct.	Nov.	Dec.	Jan.
Train A														
PTW	Temperature	°C	18.1	18.6	20.2	22.2	23.0	26.1	28.8	29.2	25.6	22.1	20.9	18.1
	BOD	mg/L	120	120	80	90	140	92	79	98	67	91	120	120
	SS	mg/L	42	35	43	34	34	36	37	32	37	37	43	45
	TN	mg/L	34.4	32.2	24.2	29.7	28.2	26.4	29.4	34.4	30.6	29.1	26.9	37.4
	TP	mg/L	3.1	2.9	1.9	2.9	3.1	2.8	2.8	2.9	2.6	2.6	2.6	3.4
STW	BOD	mg/L	3	3	6	4	5	5	2	4	2	3	12	9
	SS	mg/L	2	4	7	4	4	10	2	4	2	2	24	9
	NH4-N	mg/L	24.6	20.8	17.8	11.8	14.4	3.5	3.6	1.5	14.1	9	0.4	0.9
	NO2-N	mg/L	0.8	0.8	0.9	1.1	1.2	1.4	1.0	1.4	1.2	2.7	4.0	1.5
	NO3-N	mg/L	0.2	0.3	1.3	3.0	2.4	6.6	9.0	6.8	1.1	0.8	6.4	11.2
	TP	mg/L	0.2	0.1	0.3	0.2	0.2	0.4	0.2	0.2	0.2	0.1	0.8	0.4
AS	MLSS	mg/L	1,200	1,290	1,200	1,830	1,270	920	970	930	1,040	830	1,300	1,210
	SRT	d	4.3	4.3	2.7	4.0	3.4	2.9	2.1	2.5	3.6	2.1	3.4	5.8
Train B														
PTW	Temperature	°C	17.8	18.4	20.3	22.3	23.1	26.2	29.3	29.4	25.9	22.0	20.8	18.2
	BOD	mg/L	120	110	88	95	150	78	72	84	56	80	100	110
	SS	mg/L	55	27	43	30	33	33	37	19	36	33	39	31
	TN	mg/L	30.8	27.6	21.3	21.7	25.8	29.6	24.5	29.5	27.3	25.5	25.5	31.3
	TP	mg/L	3.3	2.8	2.2	3	3.2	2.3	2.9	3	2.5	2.1	2.5	2.9
STW	BOD	mg/L	4	5	4	4	3	4	3	5	3	3	5	7
	SS	mg/L	3	4	3	5	3	3	3	7	3	3	5	5
	NH4-N	mg/L	12.9	11.6	4.4	1.1	0.7	2.0	1.2	1.4	5.6	4.0	4.2	4.4
	NO2-N	mg/L	3.4	3.1	1.0	0.4	0.1	0.8	0.2	1.2	3.5	1.4	4.1	4.4
	NO3-N	mg/L	0.8	0.7	7.1	8.6	8.2	9.1	11.0	7.1	4.6	3.9	2.8	5.5
	TP	mg/L	0.5	0.4	0.2	0.3	0.3	0.5	0.2	0.5	0.2	0.3	0.6	0.3
AS	MLSS	mg/L	1,450	1,400	990	1,250	820	940	820	840	1,250	1,060	1,290	1,030
	SRT	d	9.3	4.4	3.6	5.9	4.6	4.2	4.7	2.0	5.2	5.2	3.9	3.9

Table S3 Complete legend for Fig. 2. (1/3)

unclassified
Patescibacteria;*Parcubacteria*;midas_o_61556;midas_f_72560;midas_g_77188;OTU01
Patescibacteria;*Parcubacteria*;midas_o_61556;midas_f_72560;midas_g_77188;(other OTUs)
Patescibacteria;*Parcubacteria*;midas_o_61556;midas_f_72560;others
Patescibacteria;*Parcubacteria*;midas_o_61556;f__;g__;OTU02
Patescibacteria;*Parcubacteria*;midas_o_61556;f__;g__;(other OTUs)
Patescibacteria;*Parcubacteria*;midas_o_61556;others
Patescibacteria;*Parcubacteria*;midas_o_22488;midas_f_45255;midas_g_54557;OTU03
Patescibacteria;*Parcubacteria*;midas_o_22488;midas_f_45255;midas_g_54557;OTU04
Patescibacteria;*Parcubacteria*;midas_o_22488;midas_f_45255;midas_g_54557;(other OTUs)
Patescibacteria;*Parcubacteria*;midas_o_22488;midas_f_45255;midas_g_45255;OTU05
Patescibacteria;*Parcubacteria*;midas_o_22488;midas_f_45255;midas_g_45255;(other OTUs)
Patescibacteria;*Parcubacteria*;midas_o_22488;midas_f_45255;others
Patescibacteria;*Parcubacteria*;midas_o_22488;others
Patescibacteria;*Parcubacteria*;midas_o_49402;midas_f_49402;g__;OTU06
Patescibacteria;*Parcubacteria*;midas_o_49402;midas_f_49402;g__;(other OTUs)
Patescibacteria;*Parcubacteria*;midas_o_49402;midas_f_49402;others
Patescibacteria;*Parcubacteria*;midas_o_49402;others
Patescibacteria;*Parcubacteria*;Ca_Vogelbacteria;midas_f_81647;midas_g_81647;OTU07
Patescibacteria;*Parcubacteria*;Ca_Vogelbacteria;midas_f_81647;midas_g_81647;(other OTUs)
Patescibacteria;*Parcubacteria*;Ca_Vogelbacteria;others
Patescibacteria;*Parcubacteria*;Ca_Nomurabacteria;midas_f_28212;midas_g_28212;OTU08
Patescibacteria;*Parcubacteria*;Ca_Nomurabacteria;midas_f_28212;midas_g_28212;(other OTUs)
Patescibacteria;*Parcubacteria*;Ca_Nomurabacteria;midas_f_28212;others
Patescibacteria;*Parcubacteria*;Ca_Nomurabacteria;midas_f_27388;midas_g_27388;OTU09
Patescibacteria;*Parcubacteria*;Ca_Nomurabacteria;midas_f_27388;midas_g_27388;(other OTUs)
Patescibacteria;*Parcubacteria*;Ca_Nomurabacteria;midas_f_46203;midas_g_77055;OTU10
Patescibacteria;*Parcubacteria*;Ca_Nomurabacteria;midas_f_46203;midas_g_77055;(other OTUs)
Patescibacteria;*Parcubacteria*;Ca_Nomurabacteria;midas_f_46203;others
Patescibacteria;*Parcubacteria*;Ca_Nomurabacteria;midas_f_28121;others
Patescibacteria;*Parcubacteria*;Ca_Nomurabacteria;f__;g__;(other OTUs)
Patescibacteria;*Parcubacteria*;Ca_Nomurabacteria;others
Patescibacteria;*Parcubacteria*;Ca_Campbellbacteria;midas_f_23156;midas_g_72638;OTU11
Patescibacteria;*Parcubacteria*;Ca_Campbellbacteria;midas_f_23156;midas_g_72638;(other OTUs)
Patescibacteria;*Parcubacteria*;Ca_Campbellbacteria;midas_f_23156;others
Patescibacteria;*Parcubacteria*;Ca_Campbellbacteria;others
Patescibacteria;*Parcubacteria*;Ca_Moranbacteria;midas_f_9621;midas_g_11532;OTU12
Patescibacteria;*Parcubacteria*;Ca_Moranbacteria;midas_f_9621;midas_g_11532;(other OTUs)
Patescibacteria;*Parcubacteria*;Ca_Moranbacteria;midas_f_9621;others
Patescibacteria;*Parcubacteria*;Ca_Moranbacteria;others
Patescibacteria;*Parcubacteria*;midas_o_24635;midas_f_24635;midas_g_32494;OTU13
Patescibacteria;*Parcubacteria*;midas_o_24635;midas_f_24635;midas_g_32494;(other OTUs)
Patescibacteria;*Parcubacteria*;midas_o_24635;midas_f_24635;others
Patescibacteria;*Parcubacteria*;o__;f__;g__;OTU14
Patescibacteria;*Parcubacteria*;o__;f__;g__;(other OTUs)
Patescibacteria;*Parcubacteria*;others
Patescibacteria;*Saccharimonadia*;*Saccharimonadales*;midas_f_5391;midas_g_6328;OTU15
Patescibacteria;*Saccharimonadia*;*Saccharimonadales*;midas_f_5391;midas_g_6328;(other OTUs)
Patescibacteria;*Saccharimonadia*;*Saccharimonadales*;midas_f_5391;midas_g_5391;OTU16
Patescibacteria;*Saccharimonadia*;*Saccharimonadales*;midas_f_5391;midas_g_5391;(other OTUs)
Patescibacteria;*Saccharimonadia*;*Saccharimonadales*;midas_f_5391;others
Patescibacteria;*Saccharimonadia*;*Saccharimonadales*;*Saccharimonadaceae*;Ca_Saccharimonas;(other OTUs)
Patescibacteria;*Saccharimonadia*;*Saccharimonadales*;*Saccharimonadaceae*;TM7a;(other OTUs)
Patescibacteria;*Saccharimonadia*;*Saccharimonadales*;*Saccharimonadaceae*;others
Patescibacteria;*Saccharimonadia*;*Saccharimonadales*;midas_f_67;others
Patescibacteria;*Saccharimonadia*;*Saccharimonadales*;midas_f_12444;midas_g_12444;OTU17
Patescibacteria;*Saccharimonadia*;*Saccharimonadales*;midas_f_12444;midas_g_12444;(other OTUs)
Patescibacteria;*Saccharimonadia*;*Saccharimonadales*;midas_f_12444;others
Patescibacteria;*Saccharimonadia*;*Saccharimonadales*;LWQ8;midas_g_12754;OTU18
Patescibacteria;*Saccharimonadia*;*Saccharimonadales*;LWQ8;midas_g_12754;(other OTUs)
Patescibacteria;*Saccharimonadia*;*Saccharimonadales*;LWQ8;others
Patescibacteria;*Saccharimonadia*;*Saccharimonadales*;midas_f_14310;others
Patescibacteria;*Saccharimonadia*;*Saccharimonadales*;midas_f_728;others
Patescibacteria;*Saccharimonadia*;*Saccharimonadales*;midas_f_3760;midas_g_10787;OTU19
Patescibacteria;*Saccharimonadia*;*Saccharimonadales*;midas_f_3760;midas_g_10787;(other OTUs)
Patescibacteria;*Saccharimonadia*;*Saccharimonadales*;midas_f_3760;others
Patescibacteria;*Saccharimonadia*;*Saccharimonadales*;others
Patescibacteria;ABY1;Ca_Magasanikbacteria;others
Patescibacteria;ABY1;others
Patescibacteria;others

Table S3 Complete legend for Fig. 2. (2/3)

Bacteroidota;OC31;midas_o_19540;midas_f_19540;midas_g_19540;OTU20

Bacteroidota;OC31;midas_o_19540;midas_f_19540;midas_g_19540;(other OTUs)

Bacteroidota;OC31;midas_o_19540;midas_f_19540;others

Bacteroidota;*Bacteroidia*;*Chitinophagales*;*Saprospiraceae*;OLB8;OTU21

Bacteroidota;*Bacteroidia*;*Chitinophagales*;*Saprospiraceae*;OLB8;(other OTUs)

Bacteroidota;*Bacteroidia*;*Chitinophagales*;*Saprospiraceae*;others

Bacteroidota;*Bacteroidia*;*Chitinophagales*;*Chitinophagaceae*;others

Bacteroidota;*Bacteroidia*;*Chitinophagales*;others

Bacteroidota;*Bacteroidia*;*Bacteroidales*;*Prevotellaceae*;others

Bacteroidota;*Bacteroidia*;*Bacteroidales*;others

Bacteroidota;*Bacteroidia*;*Sphingobacteriales*;AKYH767;others

Bacteroidota;*Bacteroidia*;*Sphingobacteriales*;env.OPS_17;midas_g_8908;OTU22

Bacteroidota;*Bacteroidia*;*Sphingobacteriales*;env.OPS_17;others

Bacteroidota;*Bacteroidia*;*Sphingobacteriales*;*Sphingobacteriaceae*;*Pedobacter*;OTU23

Bacteroidota;*Bacteroidia*;*Sphingobacteriales*;*Sphingobacteriaceae*;*Pedobacter*;(other OTUs)

Bacteroidota;*Bacteroidia*;*Sphingobacteriales*;*Sphingobacteriaceae*;others

Bacteroidota;*Bacteroidia*;*Sphingobacteriales*;others

Bacteroidota;*Bacteroidia*;Flavo*Bacteria*les;*Flavobacteriaceae*;*Flavobacterium*;OTU24

Bacteroidota;*Bacteroidia*;*Flavobacteriales*;*Flavobacteriaceae*;*Flavobacterium*;OTU25

Bacteroidota;*Bacteroidia*;*Flavobacteriales*;*Flavobacteriaceae*;*Flavobacterium*;(other OTUs)

Bacteroidota;*Bacteroidia*;*Flavobacteriales*;*Flavobacteriaceae*;others

Bacteroidota;*Bacteroidia*;*Flavobacteriales*;others

Bacteroidota;*Bacteroidia*;others

Bacteroidota;others

Campylobacterota;*Campylobacteria*;*Campylobacterales*;*Arcobacteraceae*;*Pseudarcobacter*;OTU26

Campylobacterota;*Campylobacteria*;*Campylobacterales*;*Arcobacteraceae*;*Pseudarcobacter*;(other OTUs)

Campylobacterota;*Campylobacteria*;*Campylobacterales*;*Arcobacteraceae*;*Arcobacter*;OTU27

Campylobacterota;*Campylobacteria*;*Campylobacterales*;*Arcobacteraceae*;*Arcobacter*;(other OTUs)

Campylobacterota;*Campylobacteria*;*Campylobacterales*;*Arcobacteraceae*;others

Campylobacterota;*Campylobacteria*;*Campylobacterales*;others

Actinobacteriota;*Thermoleophilia*;*Solirubrobacterales*;*Solirubrobacteraceae*;midas_g_21833;OTU28

Actinobacteriota;*Thermoleophilia*;*Solirubrobacterales*;*Solirubrobacteraceae*;midas_g_21833;(other OTUs)

Actinobacteriota;*Thermoleophilia*;*Solirubrobacterales*;*Solirubrobacteraceae*;others

Actinobacteriota;*Thermoleophilia*;*Solirubrobacterales*;others

Actinobacteriota;*Thermoleophilia*;others

Actinobacteriota;*Actinobacteria*;*Micrococcales*;*Intrasporangiaceae*;*Tetrasphaera*;(other OTUs)

Actinobacteriota;*Actinobacteria*;*Micrococcales*;*Intrasporangiaceae*;others

Actinobacteriota;*Actinobacteria*;*Micrococcales*;others

Actinobacteriota;*Actinobacteria*;others

Actinobacteriota;others

Myxococcota;*Polyangia*;*Haliangiales*;*Haliangiaceae*;*Haliangium*;OTU29

Myxococcota;*Polyangia*;*Haliangiales*;*Haliangiaceae*;*Haliangium*;OTU30

Myxococcota;*Polyangia*;*Haliangiales*;*Haliangiaceae*;*Haliangium*;(other OTUs)

Myxococcota;*Polyangia*;*Haliangiales*;*Haliangiaceae*;others

Myxococcota;*Polyangia*;mle1-27;midas_f_990;midas_g_990;(other OTUs)

Myxococcota;*Polyangia*;mle1-27;midas_f_990;others

Myxococcota;*Polyangia*;mle1-27;others

Myxococcota;*Polyangia*;*Polyangia*les;*Polyangia*ceae;others

Myxococcota;*Polyangia*;*Polyangia*les;others

Myxococcota;*Polyangia*;others

Myxococcota;others

Verrucomicrobiota;*Verrucomicrobiae*;*Verrucomicrobiales*;*Verrucomicrobiaceae*;*Prosthecobacter*;OTU31

Verrucomicrobiota;*Verrucomicrobiae*;*Verrucomicrobiales*;*Verrucomicrobiaceae*;*Prosthecobacter*;(other OTUs)

Verrucomicrobiota;*Verrucomicrobiae*;*Verrucomicrobiales*;*Verrucomicrobiaceae*;others

Verrucomicrobiota;*Verrucomicrobiae*;*Verrucomicrobiales*;Rubritaleaceae;*Luteolibacter*;OTU32

Verrucomicrobiota;*Verrucomicrobiae*;*Verrucomicrobiales*;Rubritaleaceae;*Luteolibacter*;(other OTUs)

Verrucomicrobiota;*Verrucomicrobiae*;*Verrucomicrobiales*;others

Verrucomicrobiota;*Verrucomicrobiae*;others

Verrucomicrobiota;others

Bdellovibrionota;*Oligoflexia*;0319-6G20;midas_f_4611;midas_g_38631;OTU33

Bdellovibrionota;*Oligoflexia*;0319-6G20;midas_f_4611;midas_g_38631;(other OTUs)

Bdellovibrionota;*Oligoflexia*;0319-6G20;midas_f_4611;others

Bdellovibrionota;*Oligoflexia*;0319-6G20;midas_f_3092;others

Bdellovibrionota;*Oligoflexia*;0319-6G20;others

Bdellovibrionota;*Oligoflexia*;*Silvanigrellales*;*Silvanigrellaceae*;*Silvanigrella*;OTU34

Bdellovibrionota;*Oligoflexia*;*Silvanigrellales*;*Silvanigrellaceae*;*Silvanigrella*;(other OTUs)

Bdellovibrionota;*Oligoflexia*;*Silvanigrellales*;*Silvanigrellaceae*;others

Bdellovibrionota;*Oligoflexia*;others

Bdellovibrionota

Planctomycetota

Spirochaetota;*Leptospirae*;*Leptospirales*;*Leptospiraceae*;*Turneriella*;OTU35

Table S3 Complete legend for Fig. 2. (3/3)

Spirochaetota;*Leptospirae*;*Leptospirales*;*Leptospiraceae*;*Turneriella*;(other OTUs)
Spirochaetota;*Leptospirae*;*Leptospirales*;*Leptospiraceae*;others
Spirochaetota;others
Dependentiae;*Babeliae*;*Babeliales*;others
Proteobacteria;*Alphaproteobacteria*;*Paracaedibacterales*;*Paracaedibacteraceae*;others
Proteobacteria;*Alphaproteobacteria*;*Rickettsiales*;AB1;midas_g_87794;OTU36
Proteobacteria;*Alphaproteobacteria*;*Rickettsiales*;AB1;midas_g_87794;(other OTUs)
Proteobacteria;*Alphaproteobacteria*;*Rickettsiales*;AB1;others
Proteobacteria;*Alphaproteobacteria*;*Rickettsiales*;others
Proteobacteria;*Alphaproteobacteria*;*Sphingomonadales*;*Sphingomonadaceae*;others
Proteobacteria;*Alphaproteobacteria*;*Rhodobacterales*;*Rhodobacteraceae*;others
Proteobacteria;*Alphaproteobacteria*;others
Proteobacteria;*Gammaproteobacteria*;*Competibacterales*;*Competibacteraceae*;Ca_Contendobacter;OTU37
Proteobacteria;*Gammaproteobacteria*;*Competibacterales*;*Competibacteraceae*;Ca_Contendobacter;(other OTUs)
Proteobacteria;*Gammaproteobacteria*;*Competibacterales*;*Competibacteraceae*;others
Proteobacteria;*Gammaproteobacteria*;*Enterobacterales*;*Aeromonadaceae*;*Aeromonas*;(other OTUs)
Proteobacteria;*Gammaproteobacteria*;*Enterobacterales*;*Aeromonadaceae*;*Tolumonas*;OTU38
Proteobacteria;*Gammaproteobacteria*;*Enterobacterales*;*Aeromonadaceae*;*Tolumonas*;(other OTUs)
Proteobacteria;*Gammaproteobacteria*;*Enterobacterales*;*Aeromonadaceae*;others
Proteobacteria;*Gammaproteobacteria*;*Enterobacterales*;others
Proteobacteria;*Gammaproteobacteria*;*Burkholderiales*;*Sutterellaceae*;AAP99;(other OTUs)
Proteobacteria;*Gammaproteobacteria*;*Burkholderiales*;*Sutterellaceae*;others
Proteobacteria;*Gammaproteobacteria*;*Burkholderiales*;*Comamonadaceae*;midas_g_33;(other OTUs)
Proteobacteria;*Gammaproteobacteria*;*Burkholderiales*;*Comamonadaceae*;midas_g_887;(other OTUs)
Proteobacteria;*Gammaproteobacteria*;*Burkholderiales*;*Comamonadaceae*;midas_g_191;OTU39
Proteobacteria;*Gammaproteobacteria*;*Burkholderiales*;*Comamonadaceae*;midas_g_191;(other OTUs)
Proteobacteria;*Gammaproteobacteria*;*Burkholderiales*;*Comamonadaceae*;*Aquabacterium*;OTU40
Proteobacteria;*Gammaproteobacteria*;*Burkholderiales*;*Comamonadaceae*;*Aquabacterium*;OTU41
Proteobacteria;*Gammaproteobacteria*;*Burkholderiales*;*Comamonadaceae*;*Aquabacterium*;(other OTUs)
Proteobacteria;*Gammaproteobacteria*;*Burkholderiales*;*Comamonadaceae*;*Acidovorax*;(other OTUs)
Proteobacteria;*Gammaproteobacteria*;*Burkholderiales*;*Comamonadaceae*;*Malikia*;OTU42
Proteobacteria;*Gammaproteobacteria*;*Burkholderiales*;*Comamonadaceae*;*Malikia*;(other OTUs)
Proteobacteria;*Gammaproteobacteria*;*Burkholderiales*;*Comamonadaceae*;others
Proteobacteria;*Gammaproteobacteria*;*Burkholderiales*;*Hydrogenophilaceae*;others
Proteobacteria;*Gammaproteobacteria*;*Burkholderiales*;*Neisseriaceae*;midas_g_17510;OTU43
Proteobacteria;*Gammaproteobacteria*;*Burkholderiales*;*Neisseriaceae*;midas_g_17510;OTU44
Proteobacteria;*Gammaproteobacteria*;*Burkholderiales*;*Neisseriaceae*;midas_g_17510;OTU45
Proteobacteria;*Gammaproteobacteria*;*Burkholderiales*;*Neisseriaceae*;midas_g_17510;(other OTUs)
Proteobacteria;*Gammaproteobacteria*;*Burkholderiales*;*Neisseriaceae*;others
Proteobacteria;*Gammaproteobacteria*;*Burkholderiales*;*Rhodocyclaceae*;C39;(other OTUs)
Proteobacteria;*Gammaproteobacteria*;*Burkholderiales*;*Rhodocyclaceae*;Ca_Accumulibacter;(other OTUs)
Proteobacteria;*Gammaproteobacteria*;*Burkholderiales*;*Rhodocyclaceae*;*Dechloromonas*;(other OTUs)
Proteobacteria;*Gammaproteobacteria*;*Burkholderiales*;*Rhodocyclaceae*;*Zoogloea*;(other OTUs)
Proteobacteria;*Gammaproteobacteria*;*Burkholderiales*;*Rhodocyclaceae*;others
Proteobacteria;*Gammaproteobacteria*;*Burkholderiales*;others
Proteobacteria;*Gammaproteobacteria*;*Pseudomonadales*;*Moraxellaceae*;*Agitococcus lubricus* group;OTU46
Proteobacteria;*Gammaproteobacteria*;*Pseudomonadales*;*Moraxellaceae*;*Agitococcus lubricus* group;(other OTUs)
Proteobacteria;*Gammaproteobacteria*;*Pseudomonadales*;*Moraxellaceae*;*Acinetobacter*;OTU47
Proteobacteria;*Gammaproteobacteria*;*Pseudomonadales*;*Moraxellaceae*;*Acinetobacter*;(other OTUs)
Proteobacteria;*Gammaproteobacteria*;*Pseudomonadales*;*Moraxellaceae*;*Enhydrobacter*;(other OTUs)
Proteobacteria;*Gammaproteobacteria*;*Pseudomonadales*;*Moraxellaceae*;*Alkanindiges*;OTU48
Proteobacteria;*Gammaproteobacteria*;*Pseudomonadales*;*Moraxellaceae*;*Alkanindiges*;(other OTUs)
Proteobacteria;*Gammaproteobacteria*;*Pseudomonadales*;*Moraxellaceae*;others
Proteobacteria;*Gammaproteobacteria*;*Pseudomonadales*;others
Proteobacteria;*Gammaproteobacteria*;*Xanthomonadales*;*Rhodanobacteraceae*;*Ahniella*;OTU49
Proteobacteria;*Gammaproteobacteria*;*Xanthomonadales*;*Rhodanobacteraceae*;*Ahniella*;(other OTUs)
Proteobacteria;*Gammaproteobacteria*;*Xanthomonadales*;*Rhodanobacteraceae*;*Dokdonella*;(other OTUs)
Proteobacteria;*Gammaproteobacteria*;*Xanthomonadales*;*Rhodanobacteraceae*;others
Proteobacteria;*Gammaproteobacteria*;*Xanthomonadales*;others
Proteobacteria;*Gammaproteobacteria*;others
Proteobacteria;others
Firmicutes;*Clostridia*;*Lachnospirales*;*Lachnospiraceae*;*Ruminococcus torques* group;OTU50
Firmicutes;*Clostridia*;Lachnospirales;*Lachnospiraceae*;*Ruminococcus torques* group;(other OTUs)
Firmicutes;*Clostridia*;*Lachnospirales*;*Lachnospiraceae*;others
Firmicutes;*Clostridia*;others
Firmicutes;*Bacilli*
Firmicutes;others
Chloroflexi;*Anaerolineae*;others
Chloroflexi;others
other phyla

Table S4 Temporal distribution of major OTUs across different sample types.

OTU No.	Phylogenetic Identity	Conf. Level	Temporal Distribution across Different Sample Types: Train A	Train B
OTU01	*Patescibacteria*;*Parcubacteria*;midas_o_61556; midas_f_72560;midas_g_77188;midas_s_77188	0.768		
OTU02	*Patescibacteria*;*Parcubacteria*;midas_o_61556	1.000		
OTU03	*Patescibacteria*;*Parcubacteria*;midas_o_22488; midas_f_45255;midas_g_54557	0.851		
OTU04	*Patescibacteria*;*Parcubacteria*;midas_o_22488; midas_f_45255;midas_g_54557;midas_s_63638	0.777		
OTU05	*Patescibacteria*;*Parcubacteria*;midas_o_22488; midas_f_45255;midas_g_45255;midas_s_45255	0.968		
OTU06	*Patescibacteria*;*Parcubacteria*;midas_o_49402; midas_f_49402	1.000		
OTU07	*Patescibacteria*;*Parcubacteria*;Ca_Vogelbacteria; midas_f_81647;midas_g_81647	0.714		
OTU08	*Patescibacteria*;*Parcubacteria*;Ca_Nomurabacteria; midas_f_28212;midas_g_28212;midas_s_28212	0.867		
OTU09	*Patescibacteria*;*Parcubacteria*;Ca_Nomurabacteria; midas_f_27388;midas_g_27388;midas_s_27388	1.000		
OTU10	*Patescibacteria*;*Parcubacteria*;Ca_Nomurabacteria; midas_f_46203;midas_g_77055;midas_s_77055	1.000		
OTU11	*Patescibacteria*;*Parcubacteria*;Ca_Campbellbacteria; midas_f_23156;midas_g_72638;midas_s_72638	0.986		
OTU12	*Patescibacteria*;*Parcubacteria*;Ca_Moranbacteria; midas_f_9621;midas_g_11532;midas_s_11532	0.881		
OTU13	*Patescibacteria*;*Parcubacteria*;midas_o_24635; midas_f_24635;midas_g_32494;midas_s_32494	0.998		
OTU14	*Patescibacteria*;*Parcubacteria*	1.000		
OTU15	*Patescibacteria*;*Saccharimonadia*;*Saccharimonadales*; midas_f_5391;midas_g_6328;midas_s_6328	0.899		
OTU16	*Patescibacteria*;*Saccharimonadia*;*Saccharimonadales*; midas_f_5391;midas_g_5391;midas_s_6496	0.944		
OTU17	*Patescibacteria*;*Saccharimonadia*;*Saccharimonadales*; midas_f_12444;midas_g_12444;midas_s_12444	1.000		
OTU18	*Patescibacteria*;*Saccharimonadia*;*Saccharimonadales*; LWQ8;midas_g_12754;midas_s_12754	1.000		
OTU19	*Patescibacteria*;*Saccharimonadia*;*Saccharimonadales*; midas_f_3760;midas_g_10787;midas_s_10787	0.992		
OTU20	*Bacteroidota*;OC31;midas_o_19540;midas_f_19540; midas_g_19540;midas_s_19540	1.000		
OTU21	*Bacteroidota*;*Bacteroidia*;*Chitinophagales*; *Saprospiraceae*;OLB8;midas_s_8982	1.000		
OTU22	*Bacteroidota*;*Bacteroidia*;*Sphingobacteriales*; env.OPS_17;midas_g_8908;midas_s_18546	0.838		
OTU23	*Bacteroidota*;*Bacteroidia*;*Sphingobacteriales*; *Sphingobacteriaceae*;*Pedobacter*;midas_s_12268	1.000		
OTU24	*Bacteroidota*;*Bacteroidia*;*Flavobacteriales*; *Flavobacteriaceae*;*Flavobacterium*;midas_s_9936	0.998		
OTU25	*Bacteroidota*;*Bacteroidia*;*Flavobacteriales*; *Flavobacteriaceae*;*Flavobacterium*;*Flavobacterium lutivivi*	0.988		
OTU26	Campylobacterota;Campylobacteria;Campylobacterales; Arcobacteraceae;Pseudarcobacter;midas_s_1505	0.993		
OTU27	*Campylobacterota*;*Campylobacteria*;*Campylobacterales*; *Arcobacteraceae*;*Arcobacter*;midas_s_2255	0.992		
OTU28	*Actinobacteriota*;*Thermoleophilia*;*Solirubrobacterales*; *Solirubrobacteraceae*;midas_g_21833;midas_s_56997	0.943		
OTU29	*Myxococcota*;*Polyangia*;*Haliangiales*; *Haliangiaceae*;*Haliangium*;midas_s_8233	1.000		
OTU30	*Myxococcota*;*Polyangia*;*Haliangiales*; *Haliangiaceae*;*Haliangium*;midas_s_2203	1.000		
OTU31	*Verrucomicrobiota*;*Verrucomicrobiae*;*Verrucomicrobiales*; *Verrucomicrobiaceae*;*Prosthecobacter*	0.993		
OTU32	*Verrucomicrobiota*;*Verrucomicrobiae*;*Verrucomicrobiales*; *Rubritaleaceae*;*Luteolibacter*;midas_s_55001	0.993		
OTU33	*Bdellovibrionota*;*Oligoflexia*;0319-6G20; midas_f_4611;midas_g_38631	1.000		
OTU34	*Bdellovibrionota*;*Oligoflexia*;*Silvanigrellales*; *Silvanigrellaceae*;*Silvanigrella*;midas_s_58153	0.889		
OTU35	*Spirochaetota*;*Leptospirae*;*Leptospirales*; *Leptospiraceae*;*Turneriella*;*Turneriella parva*	1.000		
OTU36	*Proteobacteria*;*Alphaproteobacteria*;*Rickettsiales*; AB1;midas_g_87794;midas_s_87794	1.000		
OTU37	*Proteobacteria*;*Gammaproteobacteria*;*Competibacterales*; *Competibacteraceae*;Ca_Contendobacter;midas_s_45613	1.000		
OTU38	*Proteobacteria*;*Gammaproteobacteria*;*Enterobacterales*; *Aeromonadaceae*;*Tolumonas*	1.000		
OTU39	*Proteobacteria*;*Gammaproteobacteria*;*Burkholderiales*; *Comamonadaceae*;midas_g_191;midas_s_28605	0.999		
OTU40	*Proteobacteria*;*Gammaproteobacteria*;*Burkholderiales*; *Comamonadaceae*;*Aquabacterium*;midas_s_77492	0.741		
OTU41	*Proteobacteria*;*Gammaproteobacteria*;*Burkholderiales*; *Comamonadaceae*;*Aquabacterium*	0.993		
OTU42	*Proteobacteria*;*Gammaproteobacteria*;*Burkholderiales*; *Comamonadaceae*;*Malikia*;midas_s_15138	0.820		
OTU43	*Proteobacteria*;*Gammaproteobacteria*;*Burkholderiales*; *Neisseriaceae*;midas_g_17510;midas_s_17510	1.000		
OTU44	*Proteobacteria*;*Gammaproteobacteria*;*Burkholderiales*; *Neisseriaceae*;midas_g_17510;midas_s_25485	0.987		
OTU45	*Proteobacteria*;*Gammaproteobacteria*;*Burkholderiales*; *Neisseriaceae*;midas_g_17510;midas_s_17510	1.000		
OTU46	*Proteobacteria*;*Gammaproteobacteria*;*Pseudomonadales*; *Moraxellaceae*;*Agitococcus lubricus* group;midas_s_11616	0.971		
OTU47	*Proteobacteria*;*Gammaproteobacteria*;*Pseudomonadales*; *Moraxellaceae*;*Acinetobacter*	1.000		
OTU48	*Proteobacteria*;*Gammaproteobacteria*;*Pseudomonadales*; *Moraxellaceae*;*Alkanindiges*	1.000		
OTU49	*Proteobacteria*;*Gammaproteobacteria*;*Xanthomonadales*; *Rhodanobacteraceae*;*Ahniella*	1.000		
OTU50	*Firmicutes*;*Clostridia*;*Lachnospirales*;*Lachnospiraceae*; *Ruminococcus torques* group;*Ruminococcus faecis*	0.990		

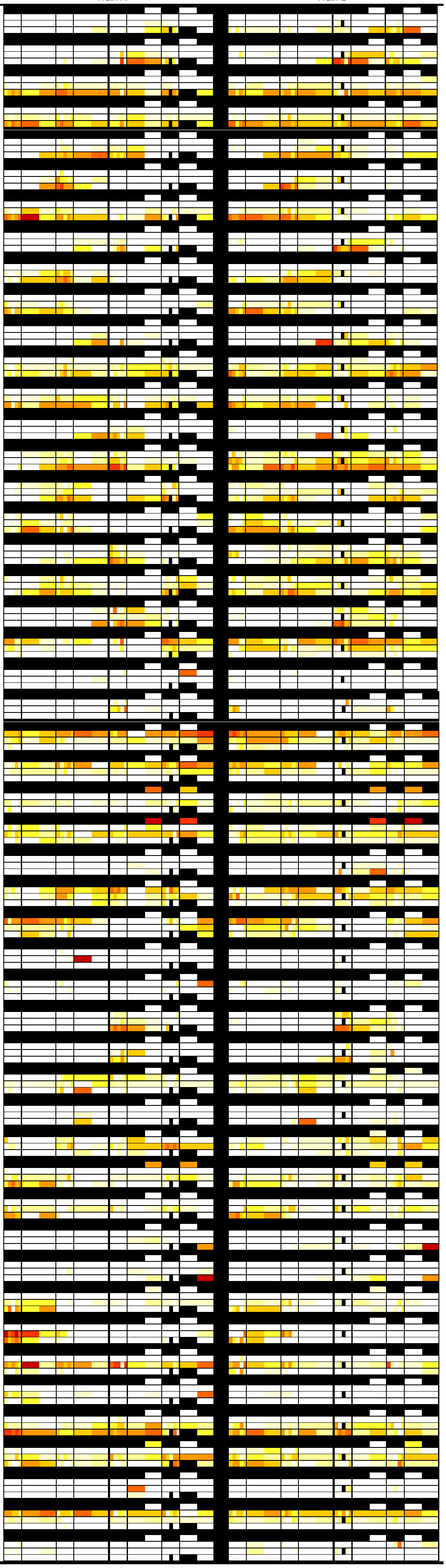

Table S5 Leak type categorization result. (1/3)

phylum class order family genus species	OTU	Read Occupancies (%) Train A PTW	Train A AS	Train A STW	Train A FSTW	Train B PTW	Train B AS	Train B STW	Train B FSTW	Leak Type Train A	Leak Type Train B
Patescibacteria		0.55	6.83	15.46	59.38	1.03	7.90	18.66	61.39	LTEF	LTEF
Parcubacteria		0	0.14	5.37	32.23	0.00	0.14	6.07	36.99	LTEF	LTEF
midas_o_61556		0	0	0.17	1.66	0	0	0.33	4.96	LTEF	LTEF
midas_f_72560		0	0	0.01	0.17	0	0	0.00	0.57	LTEF	LTEF
midas_g_77188		0	0	0.01	0.17	0	0	0.00	0.56	LTEF	LTEF
midas_s_77188	OTU01	0	0	0.01	0.17	0	0	0.00	0.56	LTEF	LTEF
	OTU02	0	0	0.10	1.03	0	0	0.21	3.14	LTEF	LTEF
midas_o_22488		0	0.01	0.63	8.18	0	0.01	0.58	9.75	LTEF	LTEF
midas_f_45255		0	0.01	0.54	7.10	0	0.01	0.41	7.26	LTEF	LTEF
midas_g_54557		0	0	0.38	5.08	0	0.00	0.32	5.25	LTEF	LTEF
	OTU03	0	0	0.16	2.70	0	0.00	0.11	1.94	LTEF	LTEF
midas_s_63638	OTU04	0	0	0.19	1.90	0	0	0.16	2.47	LTEF	LTEF
midas_g_45255		0	0.01	0.15	1.97	0	0.00	0.09	1.97	LTEF	LTEF
midas_s_45255	OTU05	0	0.01	0.10	1.22	0	0.00	0.08	1.36	LTEF	LTEF
midas_o_49402		0	0.04	0.13	1.34	0	0.00	0.14	1.53	LTEF	LTEF
midas_f_49402		0	0.04	0.12	1.34	0	0	0.14	1.53	LTEF	LTEF
	OTU06	0	0.04	0.12	1.26	0	0	0.13	1.38	LTEF	LTEF
Ca_Vogelbacteria		0	0	0.22	2.56	0	0	0.24	2.30	LTEF	LTEF
midas_f_81647		0	0	0.22	2.50	0	0	0.24	2.28	LTEF	LTEF
midas_g_81647		0	0	0.22	2.50	0	0	0.24	2.28	LTEF	LTEF
	OTU07	0	0	0.20	2.35	0	0	0.20	1.96	LTEF	LTEF
Ca_Nomurabacteria		0	0.06	2.60	10.43	0	0.01	2.35	10.02	LTEF	LTEF
midas_f_28212		0	0	0.10	0.85	0	0	0.21	2.02	LTEF	LTEF
midas_g_28212		0	0	0.10	0.84	0	0	0.21	2.02	LTEF	LTEF
midas_s_28212	OTU08	0	0	0.03	0.41	0	0	0.13	1.42	LTEF	LTEF
midas_f_27388		0	0	0.23	1.29	0	0	0.16	0.82	LTEF	LTEF
midas_g_27388		0	0	0.23	1.29	0	0	0.16	0.82	LTEF	LTEF
midas_s_27388	OTU09	0	0	0.21	1.14	0	0	0.16	0.75	LTEF	LTEF
midas_f_46203		0	0.01	0.24	0.95	0	0	0.20	0.97	LTEF	LTEF
midas_g_77055		0	0.01	0.16	0.66	0	0	0.15	0.80	LTEF	LTEF
midas_s_77055	OTU10	0	0	0.11	0.53	0	0	0.13	0.65	LTEF	LTEF
midas_f_28121		0	0	0.17	1.76	0	0	0.09	0.78	LTEF	LTEF
Ca_Campbellbacteria		0	0	0.06	0.66	0	0.00	0.14	1.09	LTEF	LTEF
midas_f_23156		0	0	0.02	0.30	0	0	0.09	0.85	LTEF	LTEF
midas_g_72638		0	0	0.01	0.27	0	0	0.09	0.82	LTEF	LTEF
midas_s_72638	OTU11	0	0	0.01	0.27	0	0	0.09	0.79	LTEF	LTEF
Ca_Moranbacteria		0	0.03	0.53	1.19	0	0.01	0.77	1.86	LTEF	LTEF
midas_f_9621		0	0.03	0.49	1.12	0	0.01	0.70	1.66	LTEF	LTEF
midas_g_11532		0	0.03	0.43	0.81	0	0.01	0.66	1.49	LTE	LTEF
midas_s_11532	OTU12	0	0.03	0.43	0.81	0	0.01	0.65	1.48	LTE	LTEF
midas_o_24635		0	0	0.31	2.51	0	0	0.22	1.67	LTEF	LTEF
midas_f_24635		0	0	0.31	2.51	0	0	0.22	1.67	LTEF	LTEF
midas_g_32494		0	0	0.28	2.41	0	0	0.20	1.56	LTEF	LTEF
midas_s_32494	OTU13	0	0	0.20	1.89	0	0	0.17	1.08	LTEF	LTEF
unclassified	OTU14	0	0	0.03	0.48	0	0	0.06	0.35	LTEF	LTEF
Saccharimonadia		0	6.24	5.67	24.15	0.26	7.25	8.08	21.49	LTF	LTF
Saccharimonadales		0	6.24	5.67	24.15	0.26	7.25	8.08	21.49	LTF	LTF
midas_f_5391		0	0.39	0.72	7.07	0	0.57	1.31	6.21	LTF	LTEF
midas_g_6328		0	0.21	0.50	5.38	0	0.45	1.10	4.99	LTEF	LTEF
midas_s_6328	OTU15	0	0.17	0.38	4.64	0	0.33	0.90	4.26	LTEF	LTEF
midas_g_5391		0	0.18	0.22	1.69	0	0.12	0.21	1.22	LTF	LTF
midas_s_6496	OTU16	0	0.12	0.14	1.20	0	0.09	0.15	0.90	LTF	LTF
Saccharimonadaceae		0	1.96	1.24	3.10	0.09	2.08	0.91	1.98	LTF	LTF
Ca_Saccharimonas		0	1.57	0.80	1.79	0	1.58	0.63	1.29	LTF	LTF
TM7a		0	0.20	0.36	0.99	0.09	0.35	0.21	0.51	LTF	LTF
midas_f_67		0	0.88	0.89	2.29	0	1.63	1.66	1.93	LTF	none
midas_f_12444		0	0.14	0.11	0.70	0	0.12	0.15	0.81	LTF	LTF
midas_g_12444		0	0.14	0.11	0.69	0	0.12	0.15	0.80	LTF	LTF
midas_s_12444	OTU17	0	0.14	0.10	0.69	0	0.12	0.14	0.79	LTF	LTF
LWQ8		0	0.28	0.39	3.62	0	0.33	0.77	2.12	LTF	LTEF
midas_g_12754		0	0.10	0.24	2.33	0	0.13	0.51	1.61	LTEF	LTEF
midas_s_12754	OTU18	0	0.07	0.17	1.45	0	0.08	0.34	0.99	LTEF	LTEF
midas_f_14310		0	0.06	0.09	0.30	0	0.05	0.48	0.73	LTF	LTE
midas_f_728		0	0.27	0.38	1.60	0.17	0.28	0.45	1.41	LTF	LTF
midas_f_3760		0	0.39	0.34	1.13	0	0.43	0.42	2.10	LTF	LTF
midas_g_10787		0	0.23	0.26	0.88	0	0.35	0.28	1.85	LTF	LTF
midas_s_10787	OTU19	0	0.19	0.18	0.68	0	0.27	0.21	1.43	LTF	LTF
ABY1		0	0.06	2.28	2.00	0.34	0.10	2.35	1.64	LTE	LTE
Ca_Magasanikbacteria		0	0.06	1.77	1.73	0.34	0.06	1.99	1.39	LTE	LTE
Bacteroidota		4.88	25.99	11.90	3.08	8.63	25.15	14.47	4.00	NLT	NLT
OC31		0	0.27	0.08	1.03	0	0.13	0.11	2.04	LTF	LTF
midas_o_19540		0	0.27	0.08	1.03	0	0.13	0.11	2.04	LTF	LTF
midas_f_19540		0	0.27	0.08	1.03	0	0.13	0.11	2.04	LTF	LTF
midas_g_19540		0	0.27	0.08	1.03	0	0.13	0.11	2.04	LTF	LTF
midas_s_19540	OTU20	0	0.25	0.07	0.89	0	0.11	0.09	1.89	LTF	LTF
Bacteroidia		4.42	25.23	11.50	1.84	7.86	24.71	13.95	1.72	NLT	NLT
Chitinophagales		0	10.90	4.25	0.94	1.71	12.64	5.45	0.76	NLT	NLT
Saprospiraceae		0	3.59	0.83	0.15	0.60	5.78	1.61	0.20	NLT	NLT
OLB8		0	1.34	0.29	0.07	0.09	2.99	0.74	0.04	NLT	NLT
midas_s_8982	OTU21	0	1.26	0.25	0.06	0	2.57	0.66	0.04	NLT	NLT
Chitinophagaceae		0	5.43	2.10	0.21	0.85	5.74	2.29	0.28	NLT	NLT
Bacteroidales		3.13	0.41	0.87	0.22	4.10	0.34	1.08	0.22	LTE	LTE-I
Prevotellaceae		0.55	0.10	0.22	0.05	1.37	0.07	0.31	0.04	LTE	LTE-I

Table S5 Leak type categorization result. (2/3)

phylum class order family genus species	OTU	Read Occupancies (%) Train A PTW	AS	STW	FSTW	Train B PTW	AS	STW	FSTW	Leak Type Train A	Train B
Sphingobacteriales		0	4.96	2.34	0.19	0.68	3.37	2.59	0.21	NLT	NLT
AKYH767		0	2.23	0.38	0.01	0.17	1.65	0.61	0.01	NLT	NLT
env.OPS_17		0	1.90	1.17	0.10	0.17	0.98	0.98	0.12	NLT	none
midas_g_8908		0	0.08	0.00	0	0	0.01	0.00	0	NLT	NLT
midas_s_18546	OTU22	0	0.08	0.00	0	0	0.01	0.00	0	NLT	NLT
Sphingobacteriaceae		0	0.06	0.43	0	0.09	0.04	0.56	0.02	LTE	LTE
Pedobacter		0	0.06	0.40	0	0	0.04	0.51	0.02	LTE	LTE
midas_s_12268	OTU23	0	0.03	0.28	0	0	0.03	0.40	0	LTE	LTE
Flavobacteriales		1.29	7.44	3.44	0.38	1.37	6.49	3.35	0.34	NLT	NLT
Flavobacteriaceae		0.46	6.34	1.89	0.27	0.17	5.36	2.25	0.26	NLT	NLT
Flavobacterium		0.46	6.32	1.86	0.27	0.17	5.34	2.21	0.26	NLT	NLT
midas_s_9936	OTU24	0	2.39	0.40	0.03	0	2.63	0.85	0.04	NLT	NLT
Flavobacterium lutivivi	OTU25	0	1.14	0.23	0	0	1.06	0.31	0	NLT	NLT
Campylobacterota		36.68	0.19	2.01	0.23	31.45	0.24	1.81	0.29	LTE-I	LTE-I
Campylobacteria		36.68	0.19	2.01	0.23	31.45	0.24	1.81	0.29	LTE-I	LTE-I
Campylobacterales		36.68	0.19	2.01	0.23	31.45	0.24	1.81	0.29	LTE-I	LTE-I
Arcobacteraceae		33.64	0.17	1.82	0.19	28.97	0.18	1.61	0.26	LTE-I	LTE-I
Pseudarcobacter		7.37	0.01	0.35	0.05	5.56	0.01	0.31	0.06	LTE-I	LTE-I
midas_s_1505	OTU26	5.53	0	0.21	0.04	3.93	0.00	0.21	0.04	LTE-I	LTE-I
Arcobacter		24.88	0.17	1.38	0.12	21.62	0.14	1.23	0.17	LTE-I	LTE-I
midas_s_2255	OTU27	20.55	0.14	1.11	0.12	17.78	0.13	1.01	0.12	LTE-I	LTE-I
Actinobacteriota		0.55	2.80	2.42	0.56	0.09	5.14	3.52	1.50	NLT	NLT
Thermoleophilia		0	0.14	0.03	0.10	0	0.06	0.13	0.55	LTF	LTEF
Solirubrobacterales		0	0.07	0.03	0.10	0	0.05	0.10	0.53	LTF	LTEF
Solirubrobacteraceae		0	0.04	0.01	0.10	0	0.01	0.05	0.52	LTF	LTEF
midas_g_21833		0	0	0.00	0.10	0	0	0.02	0.52	LTEF	LTEF
midas_s_56997	OTU28	0	0	0.00	0.01	0	0	0.02	0.50	LTEF	LTEF
Actinobacteria		0.18	1.87	1.95	0.37	0	4.14	2.78	0.89	none	NLT
Micrococcales		0.09	1.26	1.21	0.17	0	3.06	1.84	0.27	NLT	NLT
Intrasporangiaceae		0	1.19	1.11	0.17	0	2.89	1.74	0.23	NLT	NLT
Tetrasphaera		0	1.17	1.07	0.16	0	2.72	1.60	0.20	NLT	NLT
Myxococcota		0	13.92	3.33	1.71	1.37	10.19	5.26	1.33	NLT	NLT
Polyangia		0	13.35	3.15	1.59	1.37	9.78	4.99	1.26	NLT	NLT
Haliangiales		0	6.30	1.68	1.12	0.43	5.29	2.52	0.83	NLT	NLT
Haliangiaceae		0	6.30	1.68	1.12	0.43	5.29	2.52	0.83	NLT	NLT
Haliangium		0	6.24	1.62	1.09	0.43	5.22	2.40	0.83	NLT	NLT
midas_s_8233	OTU29	0	2.15	0.70	0.15	0	1.75	1.21	0.13	NLT	NLT
midas_s_2203	OTU30	0	1.54	0.17	0.14	0	1.33	0.29	0.19	NLT	NLT
mle1-27		0	2.03	0.32	0.20	0.43	1.35	0.54	0.24	NLT	NLT
midas_f_990		0	2.03	0.31	0.20	0.43	1.33	0.53	0.24	NLT	NLT
midas_g_990		0	0.43	0.07	0.01	0	0.31	0.07	0.03	NLT	NLT
Polyangiales		0	3.36	0.45	0.12	0.51	2.07	0.57	0.11	NLT	NLT
Polyangiaceae		0	2.59	0.32	0.06	0.51	1.70	0.44	0.09	NLT	NLT
Verrucomicrobiota		0.46	2.01	3.83	0.43	0.85	1.54	1.39	0.32	none	NLT
Verrucomicrobiae		0.46	1.94	3.68	0.27	0.77	1.51	1.18	0.29	none	NLT
Verrucomicrobiales		0.09	0.68	2.83	0.14	0.34	0.63	0.47	0.14	LTE	NLT
Verrucomicrobiaceae		0	0.50	2.75	0.14	0.09	0.49	0.40	0.12	LTE	NLT
Prosthecobacter		0	0.07	2.46	0.10	0	0.15	0.10	0.07	LTE	NLT
	OTU31	0	0.01	2.17	0	0	0	0	0.00	LTE	LTF
Rubritaleaceae		0	0.16	0.05	0	0	0.12	0.05	0.00	NLT	NLT
Luteolibacter		0	0.16	0.05	0	0	0.12	0.05	0.00	NLT	NLT
midas_s_55001	OTU32	0	0.10	0.01	0	0	0.05	0.02	0	NLT	NLT
Bdellovibrionota		0	1.39	1.87	8.05	0.26	1.42	2.05	8.46	LTF	LTF
Oligoflexia		0	1.14	1.05	6.69	0	1.09	1.25	7.11	LTF	LTF
0319-6G20		0	1.10	0.80	6.00	0	1.00	0.87	5.61	LTF	LTF
midas_f_4611		0	0.35	0.32	3.49	0	0.34	0.33	3.63	LTF	LTF
midas_g_38631		0	0.10	0.09	2.31	0	0.22	0.18	2.81	LTF	LTF
	OTU33	0	0.06	0.05	1.53	0	0.17	0.13	1.95	LTF	LTF
midas_f_3092		0	0.61	0.31	1.69	0	0.45	0.35	1.44	LTF	LTF
Silvanigrellales		0	0.01	0.15	0.50	0	0.02	0.27	1.26	LTEF	LTEF
Silvanigrellaceae		0	0.01	0.15	0.50	0	0.02	0.27	1.26	LTEF	LTEF
Silvanigrella		0	0	0.11	0.43	0	0.01	0.25	1.13	LTEF	LTEF
midas_s_58153	OTU34	0	0	0.11	0.35	0	0.01	0.19	0.94	LTEF	LTEF
Planctomycetota		0	1.86	0.74	0.60	0.09	1.97	1.36	0.61	NLT	NLT
Spirochaetota		0.09	0.94	1.22	1.42	0.94	0.61	1.95	0.81	none	LTE
Leptospirae		0.09	0.93	1.21	1.16	0.77	0.59	1.90	0.45	none	LTE
Leptospirales		0.09	0.93	1.21	1.16	0.77	0.59	1.90	0.45	none	LTE
Leptospiraceae		0.09	0.93	1.21	1.16	0.77	0.59	1.90	0.45	none	LTE
Turneriella		0	0.60	0.48	0.82	0.43	0.39	0.72	0.26	none	none
Turneriella parva	OTU35	0	0.35	0.18	0.36	0.17	0.19	0.30	0.10	LTF	none
Dependentiae		0	0.09	0.97	0.16	0.09	0.07	1.18	0.09	LTE	LTE
Babeliae		0	0.09	0.97	0.16	0.09	0.07	1.18	0.09	LTE	LTE
Babeliales		0	0.09	0.97	0.16	0.09	0.07	1.18	0.09	LTE	LTE
Proteobacteria		45.90	38.23	52.26	19.51	43.68	38.04	43.03	16.41	none	none
Alphaproteobacteria		1.47	5.36	5.87	2.66	2.39	6.74	6.86	3.54	none	none
Paracaedibacterales		0.18	0.26	0.66	0.04	0.09	0.10	1.02	0.03	LTE	LTE
Paracaedibacteraceae		0.18	0.26	0.66	0.04	0.09	0.10	1.02	0.03	LTE	LTE
Rickettsiales		0.09	0.32	1.46	0.64	0	0.43	1.49	1.04	LTE	LTE
AB1		0	0.03	0.19	0.24	0	0.03	0.16	0.43	LTE	LTEF
midas_g_87794		0	0	0.01	0.07	0	0	0.00	0.29	LTEF	LTEF
midas_s_87794	OTU36	0	0	0.00	0.07	0	0	0.00	0.25	LTEF	LTEF
Sphingomonadales		0.09	1.44	0.87	0.13	0.26	1.71	0.93	0.16	NLT	NLT
Sphingomonadaceae		0.09	1.44	0.87	0.13	0.26	1.71	0.93	0.16	NLT	NLT
Rhodobacterales		0.65	1.29	0.56	0.70	1.20	1.46	0.63	0.68	none	none

Table S5 Leak type categorization result. (3/3)

		Read Occupancies (%)								Leak Type	
		Train A				Train B					
phylum class order family genus species	OTU	PTW	AS	STW	FSTW	PTW	AS	STW	FSTW	Train A	Train B
Rhodobacteraceae		0.65	1.29	0.56	0.70	1.20	1.46	0.63	0.68	none	none
Gammaproteobacteria		44.42	32.88	46.31	16.84	41.28	31.30	36.08	12.81	none	none
Competibacterales		0	0.87	1.86	0.08	0.09	1.08	0.61	0.21	LTE	NLT
Competibacteraceae		0	0.87	1.86	0.08	0.09	1.08	0.61	0.21	LTE	NLT
Ca_Contendobacter		0	0.25	1.72	0.06	0.09	0.25	0.39	0.08	LTE	none
midas_s_45613	OTU37	0	0.25	1.56	0.04	0.09	0.24	0.35	0.07	LTE	none
Enterobacterales		10.51	0.61	1.85	1.54	6.24	0.26	1.36	0.92	LTE-I	LTE-I
Aeromonadaceae		6.73	0.46	1.44	1.25	4.79	0.18	1.11	0.84	LTE-I	LTE-I
Aeromonas		1.84	0.45	0.93	0.41	2.14	0.16	0.66	0.30	LTE	LTE-I
Tolumonas		4.88	0.01	0.50	0.84	2.65	0.02	0.44	0.54	LTE-I	LTE-I
	OTU38	3.50	0.01	0.29	0.44	1.37	0	0.24	0.30	LTE-I	LTE-I
Burkholderiales		27.00	23.73	35.72	8.61	25.73	23.21	27.38	6.67	none	none
Sutterellaceae		0.09	1.55	0.61	0.01	0	1.32	1.00	0.03	NLT	NLT
AAP99		0	1.55	0.61	0	0	1.29	0.99	0.03	NLT	NLT
Comamonadaceae		13.27	13.05	9.84	5.13	12.91	13.37	9.67	4.19	NLT	NLT
midas_g_33		0	1.41	0.59	0.09	0.51	1.76	0.50	0.02	NLT	NLT
midas_g_887		0.09	1.48	0.51	0.17	0.34	1.83	0.81	0.16	NLT	NLT
midas_g_191		0	0	0.49	0.72	0	0	0.44	0.51	LTE	LTE
midas_s_28605	OTU39	0	0	0.32	0.54	0	0	0.37	0.43	LTE	LTE
Aquabacterium		0	0	0.15	1.95	0.17	0.02	0.04	1.75	LTEF	LTF
midas_s_77492	OTU40	0	0	0.02	0.06	0	0	0.01	1.33	LTEF	LTEF
Aquabacterium	OTU41	0	0	0.02	1.48	0	0	0.00	0.09	LTEF	LTEF
Acidovorax		9.31	1.43	1.16	0.12	8.29	1.49	1.05	0.10	NLT	NLT
Malikia		0.09	0	0.30	0.65	0.09	0	0.22	0.38	LTEF-I	LTE-I
midas_s_15138	OTU42	0.09	0	0.18	0.41	0.09	0	0.14	0.22	LTEF-I	LTE-I
Hydrogenophilaceae		0.09	0.42	0.05	0.39	0	0.24	0.12	0.10	LTF	NLT
Neisseriaceae		0.46	0.08	10.64	0.76	0.34	0.05	3.08	0.19	LTE	LTE
midas_g_17510		0.09	0	9.63	0.74	0.09	0.01	2.45	0.14	LTE-I	LTE
midas_s_17510	OTU43	0	0	3.41	0.54	0	0	0.54	0.09	LTE	LTE
midas_s_25485	OTU44	0	0	4.56	0.07	0	0.00	1.48	0.04	LTE	LTE
midas_s_17510	OTU45	0	0	0.29	0.05	0	0	0.01	0	LTE	LTE
Rhodocyclaceae		9.86	7.40	11.68	1.32	8.63	6.77	10.93	1.46	none	none
C39		1.57	0.06	3.03	0	0.26	0.00	1.01	0.02	LTE-I	LTE-I
Ca_Accumulibacter		1.01	1.24	1.58	0.43	1.79	1.88	1.55	0.47	none	NLT
Dechloromonas		2.30	1.41	0.56	0.08	2.05	1.31	0.68	0.10	NLT	NLT
Zoogloea		1.75	3.52	5.15	0.59	2.22	2.36	5.45	0.46	none	LTE
Pseudomonadales		6.91	1.81	4.10	6.20	8.80	1.63	3.26	4.38	LTE	none
Moraxellaceae		6.08	0.48	3.25	5.91	7.78	0.30	2.43	4.06	LTE-I	LTE-I
Agitococcus lubricus group		0	0.01	0.80	4.32	0	0.02	0.69	2.59	LTEF	LTEF
midas_s_11616	OTU46	0	0.01	0.54	3.51	0	0.00	0.52	1.98	LTEF	LTEF
Acinetobacter		3.59	0.39	1.67	1.00	5.04	0.15	1.06	1.04	LTE	LTE-I
	OTU47	0.46	0.01	0.99	0.56	0.43	0.06	0.58	0.73	LTE-I	LTE
Enhydrobacter		2.49	0.02	0.04	0.01	2.74	0.06	0.06	0.02	none-I	none-I
Alkanindiges		0	0.02	0.33	0.27	0	0.00	0.14	0.11	LTE	LTE
	OTU48	0	0	0.16	0.01	0	0	0.05	0.00	LTE	LTE
Xanthomonadales		0	5.14	1.75	0.17	0.26	4.14	2.15	0.14	NLT	NLT
Rhodanobacteraceae		0	4.18	1.12	0.14	0.17	3.55	1.63	0.07	NLT	NLT
Ahniella		0	2.18	0.21	0.03	0	1.82	0.43	0.01	NLT	NLT
	OTU49	0	1.66	0.13	0.02	0	1.41	0.28	0	NLT	NLT
Dokdonella		0	1.00	0.07	0.01	0	0.61	0.13	0.01	NLT	NLT
Firmicutes		7.37	2.33	2.17	0.98	6.24	2.66	2.15	1.16	NLT	NLT
Clostridia		4.79	1.13	0.82	0.48	3.85	1.37	0.94	0.50	NLT	NLT
Lachnospirales		2.86	0.43	0.35	0.07	1.79	0.79	0.45	0.07	NLT	NLT
Lachnospiraceae		2.86	0.43	0.35	0.07	1.79	0.79	0.45	0.07	NLT	NLT
Ruminococcus torques group		0	0.01	0.02	0	0.09	0.14	0.04	0	LTE	NLT
Ruminococcus faecis	OTU50	0	0.01	0.01	0	0	0.10	0.01	0	NLT	NLT
Bacilli		1.01	0.90	1.04	0.39	0.77	1.06	0.85	0.59	none	NLT
Chloroflexi		0	0.84	0.30	0.24	0	1.60	0.92	0.34	NLT	NLT
Anaerolineae		0	0.74	0.21	0.21	0	1.25	0.76	0.30	none	NLT
Bacilli		1.01	0.90	1.04	0.39	0.77	1.06	0.85	0.59	none	NLT
Chloroflexi		0	0.84	0.30	0.24	0	1.60	0.92	0.34	NLT	NLT
Anaerolineae		0	0.74	0.21	0.21	0	1.25	0.76	0.30	none	NLT
